# Profiling of Amino Acids and Their Derivatives Biogenic Amines Before and After Antipsychotic Treatment in First-Episode Psychosis

**DOI:** 10.3389/fpsyt.2018.00155

**Published:** 2018-04-24

**Authors:** Liisa Leppik, Kärt Kriisa, Kati Koido, Kadri Koch, Kärolin Kajalaid, Liina Haring, Eero Vasar, Mihkel Zilmer

**Affiliations:** ^1^Department of Physiology, Institute of Biomedicine and Translational Medicine, University of Tartu, Tartu, Estonia; ^2^Psychiatry Clinic of Tartu University Hospital, Tartu, Estonia; ^3^Department of Biochemistry, Institute of Biomedicine and Translational Medicine, University of Tartu, Tartu, Estonia

**Keywords:** first-episode psychosis, metabolic profiling, amino acids, biogenic amines, antipsychotic treatment

## Abstract

Schizophrenia (SCH) is a heterogeneous disorder, deriving from a potential multitude of etiopathogenetic factors. During the past few years there has been an increasing interest in the role of circulating amino acids (AAs) and biogenic amines (BAs) in the pathophysiology of SCH. In the present study, we aimed to provide an insight into the potential role of alterations in levels of AAs and BAs as well as examine their more specific metabolic shifts in relation to early stage of SCH. We measured 21 AAs and 17 BAs in serum samples of patients with first-episode psychosis (FEP) before and after 7-month antipsychotic treatment in comparison to control subjects (CSs). According to multivariate analysis, antipsychotic-naïve FEP patients had significantly higher levels of taurine and spermine, whereas values of proline (Pro), alpha-aminoadipic acid (alpha-AAA), kynurenine (Kyn), valine (Val), tyrosine (Tyr), citrulline (Citr), tryptophan (Trp), and histidine (His) were diminished compared to CSs. Increased levels of taurine and spermine, as well as reduced levels of alpha-AAA and Kyn probably reflect the compromised function of *N*-methyl-D-aspartate (NMDA) receptors in patients. The decreased levels of Pro (AA modulating the function of glutamate decarboxylase) likely reflect the imbalanced function of gamma-aminobutyric acid (GABA) system in the brain of FEP patients. The alterations in ratio between Tyr and phenylalanine (Phe) can be taken as a sign of compromised function of dopaminergic system. These metabolic shifts were reinstated by 7-month antipsychotic treatment. Serum metabolic profiles can be regarded as important indicators to investigate clinical course of SCH and treatment response.

## Introduction

Schizophrenia (SCH) is a complex, heterogeneous disorder with a multitude of psychopathological, cognitive, behavioral and biological perturbations. Several hypotheses have been proposed to explain the pathological processes inherent to SCH spectrum psychoses. Disrupted brain development [1–3], disturbed neurotransmission [4, 5], imbalance in immune, oxidative and metabolic systems as well as dysregulated metabolic pathways both in the central nervous system (CNS) and periphery contribute to the pathophysiology of the SCH [6–12]. In addition to the disease manifestation in specific ways, antipsychotic drugs may play an important role in the alterations in abovementioned systems and pathways. At present, biological treatment of SCH mainly consists of antipsychotic drugs which primarily treat the traditional endpoint of disorder associated with abnormal biogenic amine signaling, and may bring along unwanted metabolic side effects. It has been suggested that therapeutic strategies which target the underlying dysfunctions in the metabolic pathways may be promising alternatives, particularly for patient stratification and personalized medicine strategies [10]. Metabolomics, involving studies of metabolites of the organisms, in biological samples, is a novel approach for exploring disease biomarkers or to identify disease or intervention related perturbed metabolic pathways [13, 14].

During the past few years there has been an increasing interest in the role of circulating amino acids (AAs) and their derivative biogenic amines (BAs) in the pathophysiology of SCH. It sounds logical that AAs themselves and their derivatives BAs might have impact in psychotic disorders. Firstly, level of AAs in the CNS depends on levels of AAs in the blood. For example, blood concentrations of isoleucine (Ile), valine (Val), leucine (Leu), and phenylalanine (Phe) influence the levels of tyrosine (Tyr) and tryptophan (Trp) in the CNS due to competition for the same carriers in the blood-brain barrier [15]. Consequently, the production of BAs [dopamine (DA), norepinephrine, serotonin and histamine] in the CNS is related both to the uptake of their precursors [Tyr, Trp, Histidine (His)] and levels of Ile, Leu, and Val in the circulation. Secondly, some AAs like glutamate (Glu), aspartate (Asp), serine (Ser), and glycine (Gly) act as the excitatory and inhibitory neurotransmitters [16]. Beside the role as neurotransmitter, Glu is implicated as the precursor in biosynthesis of γ-aminobutyric acid (GABA), a major inhibitory neurotransmitter in the CNS [17].

Several reports have targeted the levels of peripheral AAs and BAs in SCH patients. Higher concentration of Glu, homocysteine (Hcy), arginine (Arg), Ile and Val and lower levels of kynurenine (Kyn), and creatinine, are reported by many authors but the levels of other AAs and BAs show inconsistent results [18–22]. Metabolomic studies in SCH are complicated by the high symptom diversity, disease duration and treatment options. To minimize or control these confounders, the first-episode psychosis (FEP) (including antipsychotic-naïve) patients have to be evaluated. Regarding FEP patients rather limited data exist concerning the changes in levels of AAs and BAs in their blood. So far the existing studies demonstrated altered levels of Glu [23] and Hcy [24, 25] in FEP, and also higher levels of Asp, citrulline (Citr), Phe, and lower serum levels of Tyr, Trp, and the ratio of Trp to the competing AAs in drug-free (including drug-naïve) SCH patients compared to control subjects (CSs) [26]. Recently, we established significant shift in blood levels of taurine and spermine in FEP patients [27], and that levels of methionine sulfoxide (Met-So) and methionine (Met) did not differ between antipsychotic-naïve FEP patients and individuals without a history of psychiatric disorders [28]. However, with regard to serum levels of oxidative stress parameters, existing literature provides rather conflicting evidence [12].

Definitely further studies are needed by using more comprehensive approach for measurement of circulating AAs and their derivatives BAs for better understanding of the impact of their circulating levels in SCH and for shedding light on their possible usefulness as biomarkers for treatment outcome, particularly among FEP patients group. Therefore, we set up a prospective study and measured AAs and their derivatives BAs in serum samples of patients with FEP before and after 7-months antipsychotic treatment compared to age-and sex-matched CSs. The main objectives of this metabolic study were to characterize general profile alterations in both circulating AAs and BAs as well as examine more specific shifts in metabolic profiles in relation to early stage of the SCH spectrum disorders and antipsychotic treatment.

## Materials and methods

### Participants

People with psychosis (38 patients) were recruited at the time of their first clinical contact for psychotic symptoms at the Psychiatric Clinic of Tartu University Hospital, Estonia. The patients fulfilled the following inclusion criteria: men or women aged between 18 and 45; had experienced the FEP; the duration of their untreated psychosis had been <3 years; no antipsychotic use prior to the study. Participants with psychosis were allowed to receive anti-anxiety medication the night before first blood sample was drawn, but not on the day of assessment. Patients were excluded from the study if they had psychotic disorders owing to a general medical condition or had substance induced psychosis. FEP diagnoses were based on clinical interviews according to the International Classification of Diseases, Tenth Edition (ICD-10) criteria [29]. 36 FEP patients underwent treatment using antipsychotic medication; two refused and they were excluded from the follow-up analysis. History of antipsychotic medication use was collected according to reviews of patient's medical charts. No restrictions were made in terms of usage of specific pharmacological agent due to naturalistic and longitudinal study design and patients were treated with various antipsychotic medications according to clinically relevant circumstances. During the second blood collection 13 patients received quetiapine (among them 7 cases as only antipsychotic treatment), 10 patients received aripiprazole (3 cases as only antipsychotic treatment), 12 were treated with olanzapine (9 cases as only antipsychotic treatment), 2 patients were assigned to risperidone (1 case as only treatment), 2 patients to sertindole (1 case as only antipsychotic treatment), 3 patients to ziprasidone, 2 patients to clozapine (in both cases clozapine was administered in the combination with other psychotropic drugs), and 2 patients to perfenazine (1 case as only treatment). At the time of the follow-up blood collection the mean theoretical chlorpromazine dose equivalent [30] was 396 ± 154 (range 80–640) mg. Twenty seven patients were treated only with antipsychotics, but nine patients additionally needed mood stabilizers, antidepressants, or hypnotics.

The control group was recruited using advertisements and 37 mentally healthy subjects participated in the study as CSs.

We attempted to match the control group to the psychosis group on body mass index (BMI), age, gender, smoking habit (yes/no), and residence in the catchment area of the Psychiatric Clinic of Tartu University Hospital. CSs were interviewed by experienced psychiatric doctors in order to avoid the inclusion of CSs with mental disorders. Exclusion criteria for the control group also included psychotic disorders among close relatives. Additional exclusion criteria for all participants were: history of diabetes, neurological, and immune-related diseases. As it was a naturalistic study, substance abuse was not exclusion criteria for either group. This study was carried out in accordance with the recommendations of Guidelines drawn up by The Norwegian National Research Ethics Committee for medical and health research with written informed consent from all subjects. All subjects gave written informed consent in accordance with the Declaration of Helsinki. The protocol was approved by the Ethics Review Committee on Human Research of the University of Tartu (Estonia). Both FEP and CSs groups were identical and subjected to the versatile characterization by previous studies [27, 28, 31–33].

### Procedure

Blood samples, clinical and BMI data of the FEP patients were assessed at two time points: on admission and after the follow-up period (mean duration 7.18 ± 0.73 months). The time duration between two occasions consisted of initial stabilization of acute psychotic symptoms (took approximately a month) and further 6-month continuous treatment with antipsychotics. Range and severity of psychopathology was assessed using the Brief Psychiatric Rating Scale (BPRS) [34]. The BPRS consists of 18 symptoms and each item is measured on a seven-point Likert scale from “not present” to “extremely severe.” A total score was used as the outcome.

Fasting blood samples and BMI data from CSs were collected cross-sectionally.

### Blood collection and clinical laboratory measurements

Fasting blood samples of participants were collected using standard venipuncture technique between 09:00 and 11:00 a.m. Blood (5 ml) was sampled in anticoagulant-free tubes and kept for 1 h at 4°C (for platelet activation) before serum was isolated (centrifugation at 2,000 rpm for 15 min at 4°C). Serum was kept at −20°C before testing.

### Measurement of AAs and BAs

To assay serum level of AAs and BAs we applied the AbsoluteIDQ™ p180 kit (BIOCRATES Life Sciences AG, Innsbruck, Austria) using the flow injection analysis tandem mass spectrometry ([FIA]-MS/MS) as well as liquid chromatography ([LC]-MS/MS) technique. The assays were performed according to the manufacturer's manual UM-P180. Identification and quantification of AAs and BAs was achieved using multiple reactions monitoring along with internal standards. Calculation of metabolite concentrations was automatically performed by MetIDQ™ software (BIOCRATES Life Sciences AG). Data quality was checked based both on the level of detection and the level of quantification (LOD or LLOQ, see also special comments at the bottom of Supplementary Tables) and median and range of all measured AAs and BAs are given in the Tables S-1, S-2, S-4, S-5, S-7, S-8.

### Statistical analyses

All data were examined for normality of distribution using the Shapiro-Wilk test. Normally distributed data (age, BMI, psychopathology score) were analyzed using the Student's *t*-test. Non-normally distributed data (levels of the metabolic markers) were analyzed using the Mann-Whitney *U*-test to establish preliminary metabolic profile differences between-groups (FEP patient's pre-treatment or post-treatment condition vs. CSs). Wilcoxon Matched Pairs Test was used to demonstrate within group (FEP patients pre- vs. post-treatment condition) AAs and BAs levels differences. For within-subject's analyses patients were paired one by one. Because of large number of simultaneous comparisons, meaningful differences were determined by setting the significance level using the Bonferroni procedure. This resulted in a corrected critical *p*-value for the AAs and BAs between- and within-group level differences of ≤ 0.001. The effect sizes (eta^2^) for significant non-parametric test results were calculated (the values of squared standardized test statistics (Z) were divided by the total number of observations on which Z was based). Effect sizes were interpreted as small, moderate, and large, with corresponding eta-squared ranging from 0.01 to 0.05, 0.06, to 0.13, and ≥0.14, respectively [35]. Dichotomous data (gender and smoking status) were analyzed using the chi-square test.

In the next step we used general linear models (GLM) to demonstrate biomarkers level's differences between the groups (i.e., drug-naïve FEP patient vs. CS, and FEP patients after treatment vs. CSs), and within the group (i.e., drug-naïve FEP patient vs. FEP patients after treatment). Prior to analyses, the metabolic data were log^10^-transformed, in order to reduce the heterogeneity of variance commonly associated with biomarker data. Categorical (disease, gender, smoking status) and continuous (age and BMI) covariates were used in the GLM to compare biomarkers levels (dependent variables) between groups. Thereafter, to determine which type of model best fits the data, backward variable elimination was used. Each subsequent step removed the least significant variable in the model until all remaining variables had individual *p*-values smaller than 0.05. *F*-tests were used to further compare the fits of linear models and analyze significant (disease or treatment) main effects in the final models and partial eta^2^-values (the proportion of the effect in addition to error variance that is attributable to the effect) were established for the final models. Partial eta^2^-values more than 0.26 were defined as large effects [35]. The statistical analyses were performed using Statistica software (StatSoft Inc., 13th Edition) [36] for Windows.

## Results

### General description of the study groups

Clinical and demographic features of all the participants included in the study are summarized in Table 1. There were no statistically significant differences between antipsychotic-naïve FEP patients and CSs in terms of age [*t*_(73)_ = 0.49, *p* = 0.62], gender [$\chi_{\left(1\right)}^{2}$ = 1.08, *p* = 0.30], or mean (± *SD*) values of BMI [22.6 ± 2.9 and 23.0 ± 3.1, respectively; *t*_(73)_ = −0.69, *p* = 0.49]. In patients during the 7-month antipsychotic treatment psychopathology (BPRS) score decreased significantly [*t*_(35)_ = 11.47, *p* < 0.0001]. In addition, 7-month treatment caused significant increase in BMI [*t*_(35)_ = −8.07, *p* < 0.0001]. Mean BMI gain at 7-months follow-up was 3.0 kg/m^2^ (± 2.2). In addition, the difference in tobacco use was not statistically significant [$\chi_{\left(1\right)}^{2}$ = 0.05, *p* = 0.82]. Ten patients (eight of them were the same, who were current cigarette smokers), and one control subject (also cigarette smoker) had used cannabis at some point during their lifetime. None of the participants met substance dependence criteria.

**Table 1:** Demographic and clinical features of study participants.

**Demographic/clinical variable**	**Antipsychotic-naïve FEP patients (*n* = 38)**	**FEP patients after 7-month treatment (*n* = 36)**	**CS (*n* = 37)**	**Group comparison (*p*-value)**
Age, years (mean ± *SD*)	25.4 ± 5.5		24.8 ± 5.3	ns
Female/Male (*n*)	17/21		21/16	ns
Cigarette smoking (*n*, %)	8 (21%)		7 (19%)	ns
BMI (mean ± *SD*)	22.6 ± 2.9	25.6 ± 4.0		<0.0001
BPRS (mean ± *SD*)	50.8 ± 14.9	23.1 ± 12.1		<0.0001

*BMI, body mass index; BPRS, Brief Psychiatric Rating Scale; ns, non-significant*.

### Profiles of AAs and BAs in FEP patients

#### FEP induced alterations in levels of AAs and BAs

First, to clarify basic differences in AAs profile of FEP patients and CSs we used Mann-Whitney *U*-test (Table 2) and detailed results are presented in the Table S-1. From 21 circulating AAs the level of seven AAs [alanine (Ala), Citr, His, Pro, Trp, Tyr, Val] and ratio between Tyr and Phe were significantly reduced in FEP patients compared to CSs. Further, we found that the most of these AAs (Ala, Citr, His, Trp, Tyr, Val) displayed a moderate difference in effect size units (η^2^ = 0.06–0.12) although their *p*-values did not survive the Bonferroni correction (Table 2). Pro (*Z* = −3.18, *p* = 0.001) and ratio between Tyr and Phe (*Z* = −4.24, *p* < 0.0001) survived the correction and displayed a large effect (η^2^ = 0.14, and η^2^ = 0.24, respectively).

**Table 2:** Comparison of serum levels of AAs (γmoles) and BAs (γmoles) between the first-episode psychosis (FEP) patients (*n* = 38) at baseline (FEP_b_) and control subjects (CSs) (*n* = 37).

**Biomarkers**	**FEP_b_**	**CSs**	***Z*-value**	***p*-value**	**Effect size (η^2^)**
	**Median (min–max)**	**Median (min–max)**			
**AMINO ACIDS**					
Alanine (Ala)	343 (206–673)	405 (232–716)	−2.10	0.04	0.06
Citrulline (Citr)	22.4 (12.6–38.1)	27.4 (11.0–48.9)	−2.95	0.003	0.12
Histidine (His)	82.6 (61.5–106)	92.1 (58.3–138)	−2.29	0.02	0.07
Proline (Pro)	166 (83.3–381)	215 (123–479)	−3.18	**0.001**	0.14
Tryptophan (Trp)	64.8 (30.3–89.3)	73.2 (32.8–120)	−2.45	0.01	0.08
Tyrosine (Tyr)	58.6 (35.8–88.7)	63.2 (33.7–159)	−2.38	0.02	0.08
Valine (Val)	198 (112–299)	220 (126–401)	−2.17	0.03	0.06
Tyr/Phenylalanine (Phe)	0.82 (0.61–1.26)	1.03 (0.49–1.57)	−4.24	**<0.0001**	0.24
**BIOGENIC AMINES**					
Alpha-Aminoadipic-acid (alpha-AAA)	0.56 (0.25–1.34)	0.76 (0.45–1.98)	−3.27	**0.001**	0.14
Kynurenine (Kyn)	2.20 (1.39–5.42)	2.70 (1.37–3.89)	−2.91	0.004	0.11
Spermine	0.27 (0.17–0.43)	0.23 (0.16–0.28)	3.20	**0.001**	0.14
Taurine	76.5 (32.4–172)	47.1 (25.8–116)	5.56	**<0.0001**	0.41

*Z-adjusted values according to Mann-Whitney U-test (FEP_b_ compared to CSs). p-values less than or equal to 0.001 after Bonferroni correction are marked in bold. Effect sizes were interpreted as moderate and large, with corresponding eta-squared ranging from 0.06 to 0.13, and ≥ 0.14, respectively*.

Considering circulating BAs statistically significant differences between FEP patients before treatment and CSs (based on Mann-Whitney *U*-test) were found for alpha-AAA, Kyn, spermine, and taurine (Table 2, and for more detailed results see Table S-2). The levels of taurine and spermine were elevated, whereas the other two BAs demonstrated the reduced levels in patients. Bonferroni correction demonstrated that alpha-AAA (*p* = 0.001), spermine (*p* = 0.001), and taurine (*p* < 0.0001) survived this procedure and displayed a large effect (η^2^ = 0.14, η^2^ = 0.14, and η^2^ = 0.41, respectively). Trp metabolite Kyn (*p* = 0.004) did not survive correction though the effect size was moderate (η^2^ = 0.11).

To confirm the existence of significant main effects of a disease on the levels of AAs and BAs, alternative methodological approach (GLM) was applied. After utilization of GLM significant biomarkers in the model were Pro (*p* = 0.0005), alpha-AAA (*p* = 0.0006), and taurine (*p* < 0.0001) whereas Val (*p* = 0.01), Tyr (*p* = 0.01), Citr (*p* = 0.02), Trp (*p* = 0.03), His (*p* = 0.04), Kyn (*p* = 0.009), and spermine (*p* = 0.04) exhibited statistically significant differences after comparing drug-naïve FEP patients and CSs (Table S-3). In the next step we used backward variables elimination procedure to remove redundant predictors from the model. The final model displayed all of the aforementioned variables (Table 3) and demonstrated a large main effect of the disease [*F*_(10, 54)_ = 9.12, *p* < 0.0001, partial eta^2^ = 0.63]. The strongest associations were established for elevation of taurine, and reduction of Pro and alpha-AAA.

**Table 3:** Statistically significant regression coefficients (ß), confidence intervals (CI), and significance values of log_10_-transformed biomarkers levels with disease, adjusted for gender, age, body mass index, and smoking status.

**Biomarkers**	**ß**	**ß (95% CI)**	***t*-value**	***p*-value**
Citrulline (Citr)	−0.27	−0.50, −0.04	−2.38	0.02
Histidine (His)	−0.25	−0.48, −0.02	−2.15	0.04
Proline (Pro)	−0.41	-0.63, −0.18	−3.64	0.0005
Tryptophan (Trp)	−0.26	−0.49, −0.03	−2.24	0.03
Tyrosine (Tyr)	−0.29	−0.52, −0.06	−2.53	0.01
Valine (Val)	−0.29	-0.50, −0.07	−2.67	0.01
Kynurenine (Kyn)	−0.29	−0.50, −0.07	−2.69	0.009
Spermine	0.26	0.02, 0.51	2.15	0.04
Taurine	0.62	0.43, 0.82	6.42	< 0.0001
Alpha–aminoadipic–acid (alpha–AAA)	−0.36	−0.56, −0.16	−3.62	0.0006

#### Antipsychotic treatment induced alterations in levels of AAs and BAs in FEP patients

Comparison of AAs levels before and after treatment (Wilcoxon matched pairs test) with antipsychotic drugs revealed that the levels of six amino acids (Ala, His, Met, Pro, Tyr, Val) and the ratio Tyr/Phe were significantly increased after 7-month treatment (Table 4, and for more detailed results see Table S-4). Only the level of Asp was lower. Further we found that five of amino acids (Ala, Met, Tyr, Val, Asp) changes displayed a moderate effect size (η^2^ = 0.09–0.12) though their change did not survive the Bonferroni correction (Table S-4). Pro (*p* < 0.0001), His (*p* = 0.0002), and the ratio Tyr/Phe (*p* < 0.0001), survived the correction for multiple comparison and they all displayed a large effect (η^2^ = 0.24, η^2^ = 0.20, and η^2^ = 0.28, respectively).

**Table 4:** Comparison of serum levels of AAs (γmoles) and BAs (γmoles) between the first-episode psychosis (FEP) patients (*n* = 36) at baseline (before treatment with antipsychotics, FEP_b_) and after 7-month treatment (FEP_f_) (*n* = 36) with antipsychotics.

**Biomarkers**	**FEP_b_**	**FEP_f_**	***Z*-value**	***p*-value**	**Effect size (η^2^)**
	**Median (min–max)**	**Median (min–max)**			
**AMINO ACIDS**					
Alanine (Ala)	343 (206–673)	418 (294–750)	2.66	0.008	0.10
Aspartate (Asp)	38.7 (18.8–62.9)	29.0 (17.7–57.4)	2.50	0.01	0.09
Histidine (His)	82.6 (61.5–106)	93.1 (73.3–132)	3.75	**0.0002**	0.20
Methionine (Met)	7.75 (4.46–26.3)	12.5 (4.53–33.5)	2.50	0.01	0.09
Proline (Pro)	166 (83.3–381)	236 (140–362)	4.15	**<0.0001**	0.24
Tyrosine (Tyr)	58.6 (35.8–88.7)	63.3 (40.6–121)	2.99	0.003	0.12
Valine (Val)	198 (112–299)	232 (136–390)	2.92	0.003	0.12
Tyr/Phe	0.82 (0.61–1.26)	1.01 (0.77–1.44)	4.46	**<0.0001**	0.28
**BIOGENIC AMINES**					
Acetylornithine (Ac-Orn)	0.56 (0.18–1.06)	0.61 (0.24–1.47)	3.41	**0.0007**	0.16
Alpha aminoadipic acid (alpha-AAA)	0.56 (0.25–1.34)	0.81 (0.33–1.54)	3.33	**0.0009**	0.15
Carnosine	0.00 (0.00–0.13)	0.00 (0.00–0.15)	2.03	0.04	0.06
Kynurenine (Kyn)	2.20 (1.39–5.42)	2.86 (1.77–4.74)	3.59	**0.0003**	0.18
Methioninesulfoxide (Met-SO)	10.4 (2.11–24.9)	8.72 (1.69–20.3)	2.05	0.04	0.06
Spermine	0.27 (0.17–0.43)	0.19 (0.16–0.27)	2.79	0.005	0.11
Taurine	76.5 (32.4–172)	46.6 (28.2–119)	5.17	**<0.0001**	0.37
Met-SO/Methionine (Met)	1.35 (0.11–4.39)	0.66 (0.05–3.55)	2.14	0.03	0.06
Kyn/Tryptophan (Trp)	0.03 (0.02–0.08)	0.04 (0.03–0.06)	2.70	0.007	0.10

*Z-values according to Wilcoxon Matched Pairs Test (FEP_b_ compared to FEP_f_). p-values less than or equal to 0.001 after Bonferroni correction are marked in bold. Effect sizes were interpreted as moderate and large, with corresponding eta-squared ranging from 0.06 to 0.13, and ≥ 0.14, respectively*.

Among BAs antipsychotic treatment caused the significant change on the levels of acetylornithine (Ac-Orn), alpha-AAA, Kyn, Met-So, spermine and taurine (Table 4, and for more detailed results see Table S-5). Besides that the ratio between Kyn and Trp, but also between Met-So and Met were affected by 7-month antipsychotic treatment. The levels of Ac-Orn, alpha-AAA, Kyn and the ratio Kyn/Trp were significantly elevated, whereas the respective values of taurine, spermine, Met-So and ratio between Met-So and Met were apparently reduced. Bonferroni correction demonstrated that taurine (*p* < 0.0001), Ac-Orn (*p* = 0.0007), alpha-AAA (*p* = 0.0009), and Kyn (*p* = 0.0003) survived this procedure and displayed a large effect (η^2^ = 0.37, η^2^ = 0.16, η^2^ = 0.15, and η^2^ = 0.18, respectively). The others [including Met-So (η^2^ = 0.06), spermine (η^2^ = 0.11), ratio between Kyn and Trp (η^2^ = 0.10), as well as ratio between Met-So and Met (η^2^ = 0.06)] demonstrated moderate effect sizes but did not survive Bonferroni method of alpha adjustment for multiple comparisons.

A repeated measures GLM was performed, to demonstrate the main effect of the 7-month antipsychotic treatment on concentrations of the serum AAs and BAs, as well as on the BMI. A decrease over time was detected in the serum levels of taurine (*p* < 0.0001) and Asp (*p* = 0.01), whereas Pro (*p* < 0.0001), His (*p* = 0.01), Ala (*p* = 0.02), alpha-AAA (*p* = 0.02), and Kyn (*p* = 0.02) simultaneously to BMI (*p* = 0.004) demonstrated significant elevations (Table S-6). Backward elimination procedure was implemented to remove valueless variables and to establish a combination of significantly changed AAs and BAs levels. The best model [*F*_(8, 58)_ = 10.14, *p* < 0.0001] revealed the positive association of BMI with changes of Pro, Asp, His, alpha-AAA, Ala, and Kyn under the influence of antipsychotic treatment (Table 5). By contrast, the levels of taurine and spermine were negatively associated with the elevated BMI. The effect size of the treatment was large (partial eta^2^ = 0.58), which indicated that approximately 58% of multivariate variance of the dependent variables is associated with the antipsychotic treatment. The most prominent effects emerged between treatment and reduction of taurine as well as elevation of Pro.

**Table 5:** Statistically significant regression coefficients (ß), confidence intervals (CI), and significance values of log_10_-transformed biomarkers levels in first-episode patients group before treatment compared to biomarkers values measured after 7 months treatment with antipsychotics.

**Biomarkers and BMI**	**ß**	**ß (95% CI)**	***t*-value**	***p*-value**
Aspartate (Asp)	0.38	0.10, 0.66	2.75	0.008
Histidine (His)	−0.32	−0.58, −0.07	−2.51	0.01
Proline (Pro)	−0.44	−0.69, −0.20	−3.58	0.0007
Alpha–aminoadipic acid (alpha–AAA)	−0.36	−0.62, −0.09	−2.68	0.009
Kynurenine (Kyn)	−0.35	−0.59, −0.10	−2.83	0.006
Spermine	0.29	0.01, 0.56	2.06	0.04
Taurine	0.67	0.46, 0.88	6.31	<0.0001
BMI	−0.43	−0.70, −0.16	−3.17	0.002

#### Comparison of levels of AAs and BAs in FEP patients treated 7 months with antipsychotic drugs and in CSs

Most of AAs of FEP patients returned to the level of comparable to CSs after the 7-month antipsychotic treatment (Table 6, and for more detailed results see Table S-7). Only Met values were higher in treated patients. However, this difference did not survive Bonferroni correction and the effect size was moderate (η^2^ = 0.07).

**Table 6:** Comparison of serum levels of amino acids (γmoles) and biogenic amines (γmoles) between the first-episode psychosis (FEP) patients (*n* = 36) at follow-up (FEP_f_) (after 7-month treatment with antipsychotics) and control subjects (CSs) (*n* = 37).

**Biomarkers**	**FEP_f_**	**CSs**	***Z*-value**	***p*-value**	**Effect size (η^2^)**
	**Median (min–max)**	**Median (min–max)**			
**AMINO ACIDS**					
Methionine (Met)	12.5 (4.53–33.5)	9.08 (4.43–35.2)	2.27	0.02	0.07
**BIOGENIC AMINES**					
Carnosine	0.00 (0.00–0.15)	0.00 (0.00–0.12)	−2.02	0.04	0.06
Methionine-sulfoxide (Met-SO)	8.72 (1.69–20.3)	10.8 (3.04–23.1)	−2.26	0.02	0.07
Met-SO/Methionine (Met)	0.66 (0.05–3.55)	1.44 (0.16–4.29)	−2.43	0.02	0.08
Kyn/Tryptophan (Trp)	0.04 (0.03–0.06)	0.04 (0.03–0.05)	3.09	0.002	0.13

*Z-adjusted values according to Mann-Whitney U-test (FEP_f_ compared to CSs)*.

*Effect sizes were interpreted as moderate, with corresponding eta-squared ranging from 0.06 to 0.13*.

Among BAs the level of Met-So was significantly reduced (*p* = 0.02, η^2^ = 0.07) and due to these changes the ratio between Met-So and Met was significantly reduced in treated patients compared CSs (Table 6, and for more detailed results see Table S-8), and the effect size was again moderate (η^2^ = 0.08). Furthermore, the ratio between Kyn and Trp was elevated in treated patients compared to CSs (*p* = 0.002), the effect size was moderate (η^2^ = 0.13).

**Figure 1:**
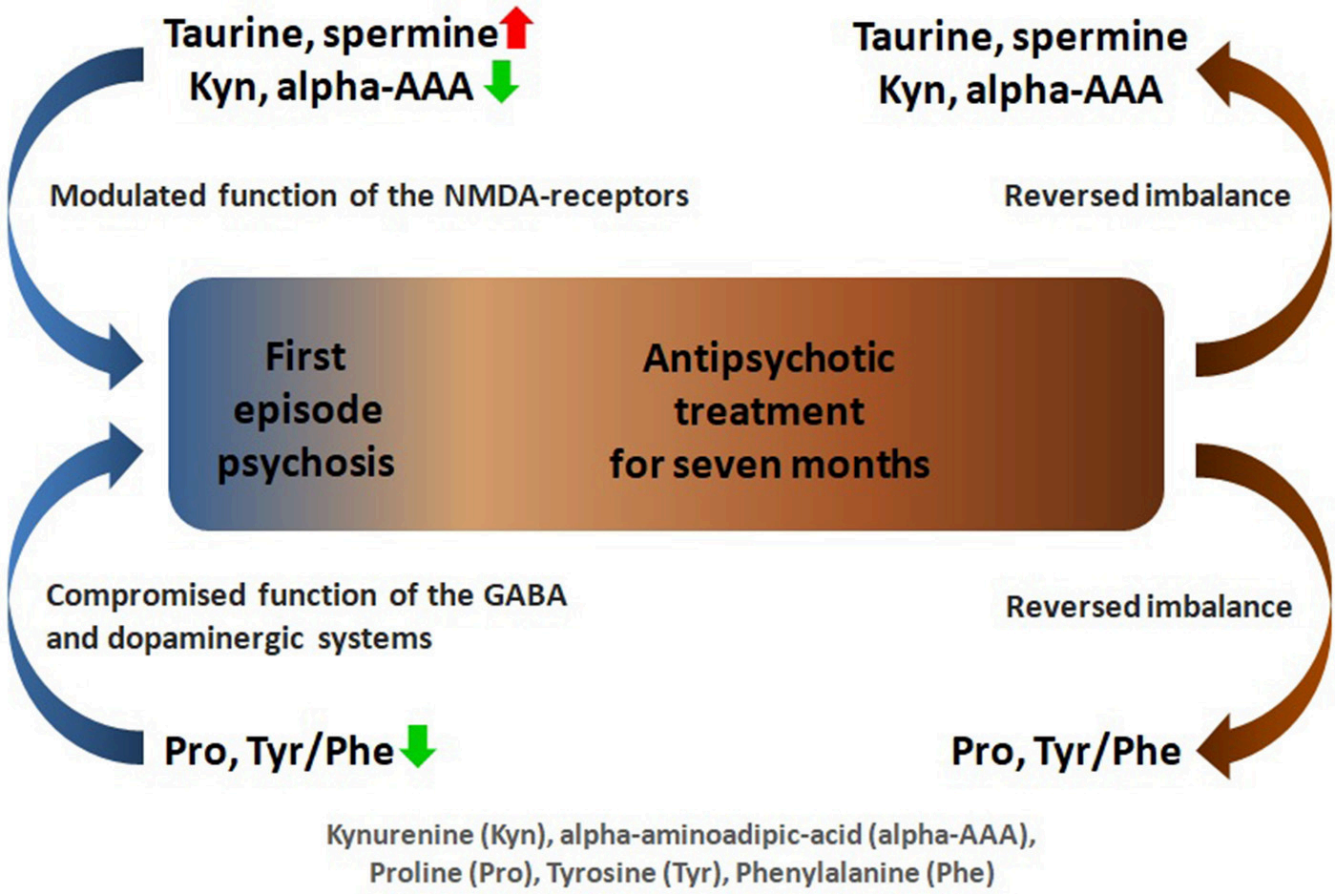
Schematic overview of amino acids and biogenic amines profiling analysis in first-episode psychosis.

Furthermore, to establish FEP patients post-treatment status in relation to AAs and BAs levels compared to CSs, GLM analyses were performed. Statistically significant differences emerged only in Gln (*p* = 0.03) and Met (*p* = 0.02) (Table S-9). Backward elimination procedure revealed that the final best model comprised Met (ß = 0.26, *t* = 2.18, *p* = 0.03) and Met-So (ß = −0.30, *t* = −2.20, *p* = 0.03). However, the whole model was not statistically significant [*F*_(2, 66)_ = 3.14, *p* = 0.05]. Figure 1 presents a schematic summary of the main findings of this study.

## Discussion

In this study, we reported altered profiles of AAs and BAs in serum of drug-naïve FEP patients and demonstrated the 7-months antipsychotic treatment impact on measured metabolite indices.

### Amino acids and biogenic amines alterations related to first psychotic episode

Performed multivariate analysis suggested that the blood levels of 6 AAs (Citr, His, Pro, Trp, Tyr, Val) and 4 BAs (alpha-AAA, Kyn, spermine and taurine) were significantly altered in drug-naïve FEP patients if compared to CSs.

Serum level of Pro was the marker most strongly affected among AAs in drug-naïve FEP patients. Pro is a one of the precursors of the neurotransmitter Glu and it serves as a modulator of synaptic transmission in the human brain [37]. An endogenous extracellular Pro regulates the basal function of some Glu synapses by retaining them in a partially potentiated state [37]. Several studies indicated that increased level of plasma Pro influence brain function in individuals with 22q11.2 deletion syndrome (22q11.2 DS) via deficiency of Pro dehydrogenase (ProDH), which gene maps to human chromosome 22, band q 11.2 [38, 39]. Individuals with 22q11.2 DS have an increased prevalence of SCH and other psychiatric conditions [40]. Furthermore, there is a report [41] that ProDH deficits produce excessive cytosolic levels of L-Pro which leads to selective dysfunction in high-frequency GABA-ergic transmission in a manner that mimics SCH. However, the conclusions with regard to studies evaluating serum Pro levels across groups of CSs, treated, drug-naïve and drug-free schizophrenic subjects have been conflicting [26, 42, 43]. In the present study, circulating levels of Pro were significantly reduced in drug-naïve FEP patients compared to CSs. It may reflect the compensatory change trying to balance the excitatory and inhibitory influences in FEP. Moreover, contradictory findings might be due to differences among samples, diagnostic subtypes or different assessment and analytical methods. In addition, given the enormous variability of the clinical manifestations and biological features of the disorder and the pleiotropic nature of most of the underlying genetic and pathophysiological mechanisms, one may suggest that the elevation of Pro may be characteristic not for FEP, but for more advanced stages of SCH.

Besides that, we demonstrated main effect of the FEP on the levels of Citr (key intermediate in the urea cycle and by-product of nitric oxide synthesis), Trp (a precursor molecule of Kyn and serotonin), His (a precursor molecule of histamine), and branched-chain amino acid Val. Val like other branched-chain amino acids Ile and Leu, is associated with abnormal glycaemic control and insulin resistance, already seen in FEP patients [44].

Furthermore, if taken alone Tyr and Phe, major precursors of catecholamine (e.g., DA) synthesis, displayed only modest changes. However, the situation was different if the ratio Tyr/Phe was calculated, which demonstrated a strong metabolic shift in favor of Phe in antipsychotic-naïve patients compared to CSs. It is noteworthy that Wei et al. [45] also showed that this ratio was significantly lower in drug-naïve male SCH patients. There is evidence suggesting the increased function of dopaminergic system in the case of positive symptoms of SCH [46]. Therefore, one may suggest the accelerated formation of DA from Tyr in dopaminergic neurons leads to reduced levels of Tyr in the circulation.

According to multivariate analysis results, elevation of taurine and spermine as well as reduction of Kyn and alpha-AAA displayed the strongest change among the studied BAs in FEP patients compared to CSs. Taurine is an aminosulphonic acid produced from sulfur containing AA cysteine (Cys) whereas Cys can be produced from Met. Thus, taurine in tightly related metabolism of Met and Cys, abundantly presented in free form in body and plays an important role in essential biological processes such as membrane stabilization, bile acid conjugation, maintenance of calcium homeostasis and osmoregulation [47]. In addition, for functioning as a neurotransmitter and an inhibitory neuromodulator in the CNS [48], taurine is potential immunomodulating compound [49, 50], it may attenuate apoptosis, to possess potent neuroprotective capacities, including inhibition of Glu-induced neurotoxicity [49], and to affect the formation of reactive oxidative species (ROS) [50]. There is remarkable evidence that some effects of taurine on ^3^H-MK801 binding, an antagonist of NMDA receptors, is apparent only in the presence of polyamine spermine [51]. Our results also indicated simultaneous and one-directional change in the levels of taurine and spermine. Spermine is formed from the non-proteining amino acid ornithine (Orn). Spermine reduces ROS-driven damages, permits correct current flow through inwardly rectifying K^+^ channels, controls activity of brain Glu receptors involved in learning and memory, and affects growth response [52]. Taurine and spermine target the GluN2B-containing NMDA receptor subtype [51, 53]. Previously, using the same cohort of participants we demonstrated strong positive link between elevated levels of taurine, spermine, epidermal growth factor (EGF) and particular inflammatory marker levels on FEP and 7-months treatment outcome [27]. Moreover, the clinical study performed by Samuelsson and colleagues (2013) [54] demonstrated significantly elevated plasma levels of taurine in patients suffering from chronic SCH and a recent research where taurine was used as the adjunctive therapy to antipsychotic treatment revealed some beneficial effects in FEP patients [55]. In addition, metabolic changes in brain taurine levels have been investigated in patients with long-term SCH. Shirayama et al. demonstrated that levels of taurine were significantly higher in the patients group than in the CSs, measured by proton magnetic resonance spectroscopy, and increased values were significantly related to the duration of illness of the patients [56]. Thus, elevated levels of taurine might offer a defense mechanisms against low-grade inflammation and oxidative stress (OxS), as well as provide compensatory function against SCH-related increases in the excitatory amino acid Glu or decreases in the inhibitory amino acid GABA.

Alpha-AAA and Kyn are examples of significantly reduced AAs derivatives in drug-naïve FEP patients in our study. Alpha-AAA is a metabolite of lysine (Lys) metabolism pathway and a marker of OxS [57, 58]. A recent metabolomic study of diabetes in human plasma samples suggested that alpha-AAA may be also modulator of glucose homeostatic imbalance and diabetes risk [59]. Alpha-AAA is a substrate of the enzyme alpha-AAA aminotransferase II, which has been shown to be the same enzyme as Kyn aminotransferase II (KAT-II), transaminating L-Kyn to KYNA [60–62]. Human brain kynurenines are not autonomous but are linked to, and influenced by, the peripheral Kyn pathway [62]. Kyn metabolites are generated by Trp catabolism and regulate biological processes that include host-microbiome signaling, immune cell response, and neuronal excitability [63]. Under the inflammatory conditions cytokines enhance indoleamine 2,3-dioxygenase 1 (IDO1) and Kyn 3-monooxygenase (K3MO) activity, and the production of 3-hydroxykynurenine (3-HK) and its metabolites [e.g., NMDA agonist quinolinic acid (QUINA)] are favored [64], which are obviously neurotoxic for both neurons and glial cells [65]. The second KAT-II-related branch produces KYNA, which has been shown to induce psychotic symptoms through antagonism at glycine-site NMDA-receptor and α-7 nicotinic acetylcholine receptors [66]. As KYNA causes hypofunction of GABAergic interneurons, disinhibition of pyramidal neurons and striatal hyper-dopaminergic status, it is considered to be involved in the pathophysiology and pathogenesis of SCH [67]. The dysregulation of Kyn level in the brain correlates with the increased or decreased activity of Kyn pathway enzymes [68]. Evidence suggests that this imbalance between KYNA and 3-HK arms in Kyn metabolism is present in medication-naïve and medication-free patients with SCH [69] as well as in the group of patients with affective psychosis [70]. Taking into account the possible interaction of AAA with Kyn metabolism one may suggest that the reduction of their levels in drug-naïve FEP patients compared to CSs may indirectly reflect the imbalance in Kyn pathways. Furthermore, altered levels of Kyn seem to be in line with our previous findings with the elevation of pro-inflammatory cytokines, EGF and taurine, which possibly reflect the corrupted function of NMDA receptors in FEP patients [27, 31].

### Impact of 7 month antipsychotic treatment on serum levels of AAs and BAs in FEP patients

Antipsychotic treatment for 7 months significantly improved the psychotic symptoms in FEP patients. However, simultaneously a significant increase of BMI occurred, demonstrating the emerging signs of metabolic syndrome. Multivariate analysis revealed that 7-month antipsychotic treatment significantly affected the levels of 3 AAs (Pro, His, and Asp) and 4 BAs (taurine, Kyn, alpha-AAA and spermine).

The elevation of circulating levels of Pro, His and the reduction of Asp were the strongest indicators of antipsychotic effect among AAs in FEP patients. Antipsychotic treatment induced reduction of Asp was exception from the other AAs, because no change of this metabolite was established in FEP patients when compared with CS. Previously, Evins et al. [71] demonstrated that clozapine treatment increased Asp serum level in patients with SCH, whereas other group [72] has reported that clozapine did not affect Asp concentration in antipsychotic-resistant schizophrenic patients. The discrepant findings with regard to Asp may be associated with different clinical phases of SCH under the studies, alternative treatment options or different duration of treatment.

In addition, the Tyr/Phe was prominently affected by antipsychotic treatment showing a strong metabolic shift in favor of Tyr if compared to Phe. This alteration could be explained by the fact that the antipsychotic drugs block DA D_2_ receptors [73], causing the increased demand and turnover of DA in the brain.

Under the treatment the levels of taurine elevated by FEP were reduced to the respective values in CSs and the similar inhibition was determined for spermine as it was established in our previous study [27]. The circulating levels of alpha-AAA and Kyn were also elevated to the level of CSs with the antipsychotic treatment.

According to the results of our study, all identified FEP induced alterations in the AAs and BAs concentrations were returned to the level of CSs after 7-months antipsychotic treatment. Multivariate analysis revealed that the final model composed of Met (ß = 0.26, *t* = 2.18, *p* = 0.03) and Met-So when treated FEP patients were compared with CSs. These changes probably reflect the inhibition of OxS in FEP patients due to the antipsychotic treatment [28].

This study has some limitations which have to be pointed out. First, the limited sample size may create generalization problems. Small cohort size in our study arose from the rarity of first episode, antipsychotic-naïve patients. We suggest that further studies including more patients with a longer follow-up period are necessary to draw firm conclusions regarding the association between treatment with antipsychotic drugs and the levels of AAs and BAs. Second, we collected data from CSs at one point in time and did not control their health condition or biomarker levels after the same follow-up period as was done for the FEP patients group. Furthermore, we did not evaluate the participant's dietary or physical activity habits. This is worth to mention, since disease chronicity and continuous antipsychotic treatment may adversely affect lifestyle factors in the group of patients with chronic psychotic disorder.

## Conclusions

Our study suggests that FEP is characterized by simultaneously elevated serum levels of taurine, spermine and diminished values of Pro, alpha-AAA, Kyn, Val, Tyr, Citr, Trp, and His if compared to CSs. Increased levels of taurine and spermine, molecules modulating activity of NMDA receptors and OxS, probably reflect the compromised function of NMDA receptors in antipsychotic-naïve FEP patients. Furthermore, the reduced levels of alpha-AAA may provide the additional support to this idea, because the reduction of alpha-AAA seems to lead to the increased level of KYNA, an antagonist of NMDA receptors. The reduced levels of Pro, AA modulating the function of Glu decarboxylase, likely reflect the altered function of GABA system in the brain of FEP patients. The alterations in ratio between Tyr and Phe can be taken as a sign of compromised function of dopaminergic system. The 7-month antipsychotic treatment may in an effective manner reverse the imbalance in AAs and BAs metabolism and that in turn manifests in clinical response. In relation to FEP, further validation of these biomarkers in larger cohorts of patients and with longer duration of follow-up would be considerably interesting.

## Author contributions

LL, KKa, KKoc, KKoi, and KKr: contributed to data collection and carried out the literature search. LH, EV, and MZ: designed the study, conducted data analysis and data interpretation, wrote the first draft of the manuscript and edited the manuscript. All authors have critically reviewed the manuscript for important intellectual content and have approved the final manuscript.

### Conflict of interest statement

The authors declare that the research was conducted in the absence of any commercial or financial relationships that could be construed as a potential conflict of interest.
