# Supplementary material for: Profiling of Amino Acids and Their Derivatives Biogenic Amines Before and After Antipsychotic Treatment in First-Episode Psychosis
Source: Front Psychiatry. 2018 Apr 24;9:155. doi: 10.3389/fpsyt.2018.00155 (PMC5928450; doi:10.3389/fpsyt.2018.00155)
Supplement: Supplementary file 1 [file Table_1.DOCX]

***Supplementary Material***

**Profiling of Amino Acids and their Derivatives Biogenic Amines Before and After Antipsychotic Treatment in First-Episode Psychosis**

Liisa Leppik^a,b*^, Kärt Kriisa^a^, Kati Koido^a^, Kadri Koch^a,b^, Kärolin Kajalaid^a,b^, Liina Haring^a,b,c^, Eero Vasar^a,c^, Mihkel Zilmer^a,c^

^a^ − Institute of Biomedicine and Translational Medicine, University of Tartu, Tartu, Estonia

^b^ − Psychiatry Clinic of Tartu University Hospital, Tartu, Estonia

^c^ − contribution of these authors has been equal

^*^ − corresponding author Liisa Leppik [liisa.leppik@kliinikum.ee](mailto:liisa.leppik@kliinikum.ee)

**Table S-1. Comparison of serum levels of amino acids (γmoles) between the first-episode psychosis (FEP) patients (n=38) at baseline (FEP_b_) and control subjects (CSs) (n=37).**

| *Amino acids* | FEP_b_ | CSs | Z-value | *p*-value |
| --- | --- | --- | --- | --- |
|  | Median  (min – max) | Median  (min – max) |  |  |
| Alanine (Ala) | 343  (206 – 673) | 405  (232 – 716) | -2.10 | 0.04 |
| Arginine (Arg) | 148  (88.0 – 216) | 152  (94.0 – 225) | -1.66 | 0.10 |
| Asparagine (Asn) | 36.80  (19.5 – 83.3) | 33.9  (15.0 – 60.4) | -0.34 | 0.73 |
| Aspartate (Asp) | 38.7  (18.8 – 62.9) | 34.2  (15.9 – 65.2) | 1.07 | 0.28 |
| Citrulline (Citr) | 22.4  (12.6 – 38.1) | 27.4  (11.0 – 48.9) | -2.95 | 0.003 |
| Glutamine (Gln) | 377  (118 – 813) | 308  (77.0 – 683) | 0.68 | 0.49 |
| Glutamate (Glu) | 210  (59.6 – 381) | 183  (114 – 550) | 0.42 | 0.67 |
| Glycine (Gly) | 274  (153 – 420) | 250  (123 – 443) | 1.48 | 0.14 |
| Histidine (His) | 82.6  (61.5 – 106) | 92.1  (58.3 – 138) | -2.29 | 0.02 |
| Isoleucine (Ile) | 85.0  (42.7 – 130) | 85.4  (50.1 – 179) | -0.56 | 0.57 |
| Leucine (Leu) | 165  (73.0 – 273) | 166  (79.6 – 409) | -0.16 | 0.87 |
| Lysine (Lys) | 184  (117 – 279) | 202  (107 – 309) | -1.35 | 0.18 |
| Methionine (Met) | 7.75  (4.46 – 26.3) | 9.08  (4.43 – 35.2) | 0.79 | 0.43 |
| Ornithine (Orn) | 57.3  (30.7 – 115) | 56.8  (23.4 – 91.4) | 0.56 | 0.57 |
| Phenylalanine (Phe) | 72.0  (41.8 – 101) | 67.1  (38.2 – 115) | 0.73 | 0.47 |
| Proline (Pro) | 166  (83.3 – 381) | 215  (123 – 479) | -3.18 | **0.001** |
| Serine (Ser) | 171  (99.4 – 293) | 160  (69.3 – 363) | 0.92 | 0.36 |
| Threonine (Thr) | 140  (84.7 – 214) | 154  (74.1 – 373) | -1.65 | 0.10 |
| Tryptophan (Trp) | 64.75  (30.3 – 89.3) | 73.2  (32.8 – 120) | -2.45 | 0.01 |
| Tyrosine (Tyr) | 58.6  (35.8 – 88.7) | 63.2  (33.7 – 159) | -2.38 | 0.02 |
| Valine (Val) | 198  (112 – 299) | 220  (126 – 401) | -2.17 | 0.03 |
| Citr/Arg | 0.16  (0.09 – 0.31) | 0.16  (0.08 – 0.33) | -1.35 | 0.18 |
| Tyr/Phe | 0.82  (0.61 – 1.26) | 1.03  (0.49 – 1.57) | -4.24 | **<0.0001** |

Z-adjusted values according to Mann-Whitney *U*-test (FEP_b_ compared to CSs). *p-*values less than or equal to 0.001 after Bonferroni correction are marked in bold. Commentary: all measured values are higher than LLOQ.
