# Supplementary material for: Profiling of Amino Acids and Their Derivatives Biogenic Amines Before and After Antipsychotic Treatment in First-Episode Psychosis
Source: Front Psychiatry. 2018 Apr 24;9:155. doi: 10.3389/fpsyt.2018.00155 (PMC5928450; doi:10.3389/fpsyt.2018.00155)
Supplement: Supplementary file 2 [file Table_2.DOCX]

***Supplementary Material***

**Profiling of Amino Acids and their Derivatives Biogenic Amines Before and After Antipsychotic Treatment in First-Episode Psychosis**

Liisa Leppik^a,b*^, Kärt Kriisa^a^, Kati Koido^a^, Kadri Koch^a,b^, Kärolin Kajalaid^a,b^, Liina Haring^a,b,c^, Eero Vasar^a,c^, Mihkel Zilmer^a,c^

^a^ − Institute of Biomedicine and Translational Medicine, University of Tartu, Tartu, Estonia

^b^ − Psychiatry Clinic of Tartu University Hospital, Tartu, Estonia

^c^ − contribution of these authors has been equal

^*^ − corresponding author Liisa Leppik [liisa.leppik@kliinikum.ee](mailto:liisa.leppik@kliinikum.ee)

**Table S-2. Comparison of serum levels of biogenic amines (γmoles) between the the first-episode psychosis (FEP) patients (n=38) at baseline (FEP_b_) and control subjects (CSs) (n=37).**

| *Biogenic amines* | FEP_b_ | CSs | Z-value | *p*-value |
| --- | --- | --- | --- | --- |
|  | Median  (min – max) | Median  (min – max) |  |  |
| Acetylornithine (Ac-Orn) | 0.56  (0.18 – 1.06) | 0.59  (0.18 – 2.03) | -1.44 | 0.15 |
| Asymmetric  dimethylarginine (ADMA) | 0.43  (0.30 – 0.67) | 0.43  (0.19 – 0.60) | -0.50 | 0.62 |
| Alpha-Aminoadipic-acid (alpha-AAA) | 0.56  (0.25 – 1.34) | 0.76  (0.45 – 1.98) | -3.27 | **0.001** |
| c4-OH-Pro | 0.25  (0.00 – 0.34) | 0.00  (0.00 – 0.39) | 1.79 | 0.07 |
| Carnosine | 0.00  (0.00 – 0.13) | 0.00  (0.00 – 0.12) | -0.66 | 0.51 |
| Creatinine | 69.65  (42.3 – 123) | 68.5  (35.0 – 112) | 0.37 | 0.72 |
| l-DOPA | 0.12  (0.00 – 0.26) | 0.15  (0.00 – 0.26) | -0.71 | 0.48 |
| Kynurenine (Kyn) | 2.20  (1.39 – 5.42) | 2.70  (1.37 – 3.89) | -2.91 | 0.004 |
| Histamine | 0.45  (0.37 – 0.46) | 0.38  (0.37 – 0.46) | 1.56 | 0.12 |
| Methionine-sulfoxide  (Met-SO) | 10.4  (2.11 – 24.9) | 10.8  (3.04 – 23.1) | -0.98 | 0.33 |
| Putrescine | 0.07  (0.02 – 0.19) | 0.08  (0.03 – 0.20) | -1.61 | 0.11 |
| Symmetric-dimethylarginine (S-DMA) | 0.57  (0.39 – 0.93) | 0.53  (0.39 – 0.81) | 1.22 | 0.22 |
| Serotonin (5-HT) | 0.57  (0.08 – 1.69) | 0.65  (0.19 – 1.47) | -0.92 | 0.36 |
| Spermine | 0.27  (0.17 – 0.43) | 0.23  (0.16 – 0.28) | 3.20 | **0.001** |
| t4-OH-Pro | 0.42  (0.00 – 15.30) | 0.61  (0.00 – 20.70) | -0.70 | 0.49 |
| Taurine | 76.5  (32.4 – 172) | 47.1  (25.8 – 116) | 5.56 | **<0.0001** |
| total-DMA | 0.70  (0.49 – 1.08) | 0.73  (0.37 – 0.96) | 0.39 | 0.70 |
| Met-SO/  Methionine (Met) | 1.35  (0.11 – 4.39) | 1.44  (0.16 – 4.29) | -0.86 | 0.39 |
| Kyn/  Tryptophan (Trp) | 0.03  (0.02 – 0.08) | 0.04  (0.03 – 0.05) | -1.21 | 0.22 |
| 5-HT/Trp | 0.01  (0.00 – 0.03) | 0.01  (0.00 – 0.02) | 0.27 | 0.79 |

Z-adjusted values according to Mann-Whitney *U*-test (FEP_b_ compared to CSs). *p-*values less than or equal to 0.001 after Bonferroni correction are marked in bold. Commentary: ADMA, creatinine, Kyn, Met-So, 5-HT, spermine, taurine, and total-DMA values are higher than LLOQ. Ac-Orn, alpha-AAA, histamine, S-DMA values were at least 1.5 to 3 times higher than LOD.
