# Supplementary material for: Profiling of Amino Acids and Their Derivatives Biogenic Amines Before and After Antipsychotic Treatment in First-Episode Psychosis
Source: Front Psychiatry. 2018 Apr 24;9:155. doi: 10.3389/fpsyt.2018.00155 (PMC5928450; doi:10.3389/fpsyt.2018.00155)
Supplement: Supplementary file 3 [file Table_3.DOCX]

***Supplementary Material***

**Profiling of Amino Acids and their Derivatives Biogenic Amines Before and After Antipsychotic Treatment in First-Episode Psychosis**

Liisa Leppik^a,b*^, Kärt Kriisa^a^, Kati Koido^a^, Kadri Koch^a,b^, Kärolin Kajalaid^a,b^, Liina Haring^a,b,c^, Eero Vasar^a,c^, Mihkel Zilmer^a,c^

^a^ − Institute of Biomedicine and Translational Medicine, University of Tartu, Tartu, Estonia

^b^ − Psychiatry Clinic of Tartu University Hospital, Tartu, Estonia

^c^ − contribution of these authors has been equal

^*^ − corresponding author Liisa Leppik [liisa.leppik@kliinikum.ee](mailto:liisa.leppik@kliinikum.ee)

**Table S-3. Main effect of the disease on serum levels of amino acids and biogenic amines (drug-naïve first-episode patients compared to control subjects).**

| *Biomarkers* | ß | ß (95 % CI) | *t-value* | *p*-value |
| --- | --- | --- | --- | --- |
| Alanine (Ala) | -0.21 | -0.46, 0.03 | -1.74 | 0.09 |
| Arginine (Arg) | -1.43 | -0.04, 0.01 | -0.46 | **0.03** |
| Asparagine (Asn) | 0.47 | -0.03, 0.04 | -0.43 | 0.07 |
| Aspartate (Asp) | 1.02 | -0.02, 0.05 | -0.18 | 0.30 |
| Citrulline (Citr) | -0.27 | -0.50, -0.04 | -2.38 | **0.02** |
| Glutamine (Gln) | 0.12 | -0.13, 0.37 | 0.99 | 0.33 |
| Glutamate (Glu) | -0.05 | -0.28, 0.18 | -0.46 | 0.65 |
| Glycine (Gly) | 0.20 | -0.05, 0.45 | 1.60 | 0.12 |
| Histidine (His) | -0.25 | -0.48, -0.02 | -2.15 | **0.04** |
| Isoleucine (Ile) | -0.14 | -0.36, 0.07 | -1.35 | 0.18 |
| Leucine (Leu) | -0.08 | -0.29, 0.13 | -0.79 | 0.43 |
| Lysine (Lys) | -0.13 | -0.37, 0.12 | -1.04 | 0.30 |
| Methionine (Met) | 0.06 | -0.17, 0.29 | 0.51 | 0.61 |
| Ornithine (Orn) | 0.11 | -0.13, 0.35 | 0.91 | 0.36 |
| Phenylalanine (Phe) | 0.08 | -0.16, 0.32 | 0.65 | 0.52 |
| Proline (Pro) | -0.41 | -0.63, -0.18 | -3.64 | **0.0005** |
| Serine (Ser) | 0.13 | -0.12, 0.38 | 1.05 | 0.30 |
| Threonine (Thr) | -0.19 | -0.44, 0.05 | -1.58 | 0.12 |
| Tryptophan (Trp) | -0.26 | -0.49, -0.03 | -2.24 | **0.03** |
| Tyrosine (Tyr) | -0.29 | -0.52, -0.06 | -2.53 | **0.01** |
| Valine (Val) | -0.29 | -0.50, -0.07 | -2.67 | **0.01** |
| Acetylornithine (Ac-Orn) | -0.17 | -0.42, 0.08 | -1.37 | 0.18 |
| Asymmetric dimethylarginine(ADMA) | 0.04 | -0.21, 0.29 | 0.34 | 0.73 |
| Alpha-aminoadipic-acid (Alpha-AAA) | -0.36 | -0.56, -0.16 | -3.62 | **0.0006** |
| Creatinine | 0.07 | -0.14, 0.28 | 0.69 | 0.50 |
| Kynurenine (Kyn) | -0.29 | -0.50, -0.07 | -2.69 | **0.009** |
| Histamine | 0.18 | -0.06, 0.43 | 1.48 | 0.14 |
| Methioninesulfoxide(Met-SO) | -0.12 | -0.36, 0.13 | -0.96 | 0.34 |
| Putrescine | -0.21 | -0.45, 0.03 | -1.73 | 0.09 |
| Symmetric-dimethylarginine (S-DMA) | 0.17 | -0.08, 0.42 | 1.36 | 0.18 |
| Serotonin (5-TH) | -0.05 | -0.30, 0.20 | -0.39 | 0.70 |
| Spermine | 0.26 | 0.02, 0.51 | 2.15 | **0.04** |
| Taurine | 0.62 | 0.43, 0.82 | 6.42 | **<0.0001** |
| total-DMA | 0.10 | -0.15, 0.35 | 0.79 | 0.43 |

ß – regression coefficients, CI – confidence intervals, *p*-values (derived from GLM analysis) – significance values of log_10_-transformed amino acids and biogenic amines levels with disease, adjusted for gender, smoking status, age and BMI. Significant *t*-values (*p* < 0.05) are marked in bold.

**Table S-4. Comparison of serum levels of amino acids (γmoles) between the first-episode psychosis (FEP) patients (n=36) at baseline (before treatment with antipsychotics, FEP_b_) and after 7-month treatment (FEP_f_) (n=36) with antipsychotics.**

| *Amino acids* | FEP_b_ | FEP_f_ | Z-value | *p*-value |
| --- | --- | --- | --- | --- |
|  | Median  (min–max) | Median  (min–max) |  |  |
| Alanine (Ala) | 342.5  (206.00 – 673.00) | 418  (294 – 750) | 2.66 | 0.008 |
| Arginine (Arg) | 148  (88.0 – 216) | 153  (93.0 – 218) | 0.44 | 0.66 |
| Asparagine (Asn) | 36.8  (19.5 – 83.3) | 37.9  (17.7 – 75.5) | 1.21 | 0.23 |
| Aspartate (Asp) | 38.7  (18.8 – 62.9) | 29.0  (17.7 – 57.4) | 2.50 | 0.01 |
| Citrulline (Citr) | 22.4  (12.6 – 38.1) | 24.6  (15.5 – 38.7) | 1.54 | 0.12 |
| Glutamine (Gln) | 377  (118 – 813) | 372.5  (162 – 810) | 1.52 | 0.13 |
| Glutamate (Glu) | 210  (59.6 – 381) | 207  (57.2 – 498) | 1.17 | 0.24 |
| Glycine (Gly) | 274  (153 – 420) | 267  (149 – 597) | 0.23 | 0.82 |
| Histidine (His) | 82.6  (61.5 – 106) | 93.1  (73.3 – 132) | 3.75 | **0.0002** |
| Isoleucine (Ile) | 85.0  (42.7 – 130) | 94.9  (43.9 – 190) | 1.78 | 0.07 |
| Leucine (Leu) | 165  (73.0 – 273) | 173  (85.5 – 364) | 0.41 | 0.68 |
| Lysine (Lys) | 183.5  (117 – 279) | 207  (103 – 306) | 1.47 | 0.14 |
| Methionine (Met) | 7.75  (4.46 – 26.3) | 12.5  (4.53 – 33.5) | 2.50 | 0.01 |
| Ornithine (Orn) | 57.3  (30.7 – 115) | 57.5  (28.4 – 91.9) | 0.90 | 0.37 |
| Phenylalanine (Phe) | 72.0  (41.8 – 101) | 65.6  (38.2 – 108) | 1.35 | 0.18 |
| Proline (Pro) | 166  (83.3 – 381) | 236  (140 – 362) | 4.15 | **<0.0001** |
| Serine (Ser) | 171  (99.4 – 293) | 158  (115 – 246) | 1.18 | 0.24 |
| Threonine (Thr) | 140  (84.7 – 214) | 148  (71.0 – 280) | 1.71 | 0.09 |
| Tryptophan (Trp) | 64.8  (30.3 – 89.3) | 70.5  (34.2 – 121) | 1.68 | 0.09 |
| Tyrosine (Tyr) | 58.6  (35.8 – 88.7) | 63.3  (40.6 – 121) | 2.99 | 0.003 |
| Valine (Val) | 198  (112 – 299) | 232  (136 – 390) | 2.92 | 0.003 |
| Citr/Arg | 0.16  (0.09 – 0.31) | 0.17  (0.10 – 0.31) | 1.81 | 0.07 |
| Tyr/Phe | 0.82  (0.61 – 1.26) | 1.01  (0.77 – 1.44) | 4.46 | **<0.0001** |

Z-values according to Wilcoxon Matched Pairs Test (FEP_b_ compared to FEP_f_). *p-*values less than or equal to 0.001 after Bonferroni correction are marked in bold.

Commentary: all measured values are higher than LLOQ.

**Table S-5. Comparison of serum levels of biogenic amines (γmoles) between the first-episode psychosis (FEP) patients (n=36) at baseline (FEP_b_) (before treatment with antipsychotic) and after 7-month treatment (FEP_f_) (n=36) with antipsychotics.**

| *Biogenic amines* | FEP_b_ | FEP_f_ | Z-value | *p*-value |
| --- | --- | --- | --- | --- |
|  | Median  (min – max) | Median  (min – max) |  |  |
| Acetylornithine  (Ac-Orn) | 0.56  (0.18 – 1.06) | 0.61  (0.24 – 1.47) | 3.41 | **0.0007** |
| Asymmetric  dimethylarginine (ADMA) | 0.43  (0.30 – 0.67) | 0.41  (0.29 – 0.61) | 0.20 | 0.84 |
| Alpha aminoadipic acid (alpha-AAA) | 0.56  (0.25 – 1.34) | 0.81  (0.33 – 1.54) | 3.33 | **0.0009** |
| c4-OH-Pro | 0.25  (0.00 – 0.34) | 0.00  (0.00 – 0.38) | 1.72 | 0.09 |
| Carnosine | 0.00  (0.00 – 0.13) | 0.00  (0.00 – 0.15) | 2.03 | 0.04 |
| Creatinine | 69.7  (42.3 – 123) | 71.7  (45.7 – 124) | 0.03 | 0.98 |
| l-DOPA | 0.12  (0.00 – 0.26) | 0.14  (0.00 – 0.30) | 1.05 | 0.30 |
| Kynurenine (Kyn) | 2.20  (1.39 – 5.42) | 2.86  (1.77 – 4.74) | 3.59 | **0.0003** |
| Histamine | 0.45  (0.37 – 0.46) | 0.38  (0.37 – 0.45) | 1.89 | 0.06 |
| Methioninesulfoxide  (Met-SO) | 10.4  (2.11 – 24.9) | 8.72  (1.69 – 20.3) | 2.05 | 0.04 |
| Putrescine | 0.07  (0.02 – 0.19) | 0.07  (0.03 – 0.21) | 0.07 | 0.94 |
| Symmetric-dimethylarginine  (S-DMA) | 0.57  (0.39 – 0.93) | 0.52  (0.39 – 0.80) | 1.34 | 0.18 |
| Serotonin (5-HT) | 0.57  (0.08 – 1.69) | 0.58  (0.05 – 1.33) | 1.56 | 0.12 |
| Spermine | 0.27  (0.17 – 0.43) | 0.19  (0.16 – 0.27) | 2.79 | 0.005 |
| t4-OH-Pro | 0.42  (0.00 – 15.30) | 0.60  (0.00 – 27.10) | 0.51 | 0.61 |
| Taurine | 76.5  (32.4 – 172) | 46.6  (28.2 – 119) | 5.17 | **<0.0001** |
| total-DMA | 0.70  (0.49 – 1.08) | 0.73  (0.52 – 0.98) | 0.50 | 0.62 |
| Met-SO/  Methionine (Met) | 1.35  (0.11 – 4.39) | 0.66  (0.05 – 3.55) | 2.14 | 0.03 |
| Kyn/Tryptophan (Trp) | 0.03  (0.02 – 0.08) | 0.04  (0.03 – 0.06) | 2.70 | 0.007 |
| 5-HT/Trp | 0.01  (0.00 – 0.03) | 0.01  (0.00 – 0.02) | 1.17 | 0.24 |

Z-values according to Wilcoxon Matched Pairs Test (FEP_b_ compared to FEP_f_). *p-*values less than or equal to 0.001 after Bonferroni correction are marked in bold.

Commentary: ADMA, creatinine, Kyn, Met-So, 5-HT, spermine, taurine, and total-DMA values are higher than LLOQ. Ac-Orn, alpha-AAA, histamine, S-DMA values were at least 1.5 to 3 times higher than LOD.

**Table S-6. Main effect of the antipsychotic treatment on serum levels of amino acids and biogenic amines and body mass index (BMI), first-episode psychosis patients data compared before and after 7-month treatment with antipsychotics.**

| *Biomarkers and BMI* | ß | ß (95 % CI) | *t-value* | *p*-value |
| --- | --- | --- | --- | --- |
| Alanine (Ala) | -0.35 | -0.63, -0.06 | -2.45 | **0.02** |
| Arginine (Arg) | 0.05 | -0.30, 0.40 | 0.31 | 0.76 |
| Asparagine (Asn) | -0.05 | -0.39, 0.29 | -0.29 | 0.78 |
| Aspartate (Asp) | 0.42 | 0.10, 0.75 | 2.60 | **0.01** |
| Citrulline (Citr) | -0.11 | -0.44, 0.22 | -0.66 | 0.51 |
| Glutamine (Gln) | -0.19 | -0.52, 0.14 | -1.16 | 0.25 |
| Glutamate (Glu) | 0.16 | -0.16, 0.49 | 1.00 | 0.32 |
| Glycine (Gly) | 0.03 | -0.32, 0.37 | 0.15 | 0.88 |
| Histidine (His) | -0.39 | -0.69, -0.09 | -2.60 | **0.01** |
| Isoleucine (Ile) | -0.17 | -0.51, 0.17 | -1.00 | 0.32 |
| Leucine (Leu) | -0.07 | -0.41, 0.28 | -0.38 | 0.70 |
| Lysine (Lys) | -0.05 | -0.40, 0.29 | -0.32 | 0.75 |
| Methionine (Met) | -0.21 | -0.55, 0.12 | -1.30 | 0.20 |
| Ornithine (Orn) | 0.15 | -0.20, 0.49 | 0.86 | 0.40 |
| Phenylalanine (Phe) | 0.13 | -0.21, 0.48 | 0.78 | 0.44 |
| Proline (Pro) | -0.55 | -0.79, -0.31 | -4.55 | **0.00004** |
| Serine (Ser) | 0.22 | -0.11, 0.54 | 1.35 | 0.18 |
| Threonine (Thr) | -0.16 | -0.50, 0.19 | -0.92 | 0.36 |
| Tryptophan (Trp) | -0.15 | -0.49, 0.19 | -0.90 | 0.37 |
| Tyrosine (Tyr) | -0.29 | -0.61, 0.03 | -1.84 | 0.07 |
| Valine (Val) | -0.26 | -0.58, 0.05 | -1.67 | 0.10 |
| Acetylornithine (Ac-Orn) | -0.10 | -0.45, 0.24 | -0.61 | 0.54 |
| Asymmetric dimethylarginine (ADMA) | 0.14 | -0.20, 0.48 | 0.82 | 0.41 |
| Alpha-Aminoadipic-acid (alpha-AAA) | -0.39 | -0.70, -0.08 | -2.51 | **0.02** |
| Creatinine | 0.09 | -0.24, 0.42 | 0.54 | 0.59 |
| L-DOPA | 0.09 | -0.24, 0.42 | 0.54 | 0.59 |
| Kynurenine (Kyn) | -0.35 | -0.65, -0.06 | -2.40 | **0.02** |
| Histamine | 0.21 | -0.13, 0.54 | 1.25 | 0.22 |
| Methionine-sulfoxide (Met-SO) | 0.14 | -0.19, 0.48 | 0.84 | 0.40 |
| Putrescine | 0.12 | -0.22, 0.46 | 0.70 | 0.49 |
| Symmetric-dimethylarginine (S-DMA) | 0.26 | -0.08, 0.60 | 1.56 | 0.13 |
| Serotonin (5-HT) | 0.13 | -0.20, 0.47 | 0.79 | 0.43 |
| Spermine | 0.25 | -0.08, 0.58 | 1.52 | 0.14 |
| Taurine | 0.70 | 0.46, 0.94 | 5.88 | **0.0000005** |
| total-DMA | 0.02 | -0.32, 0.37 | 0.13 | 0.90 |
| BMI | -0.48 | -0.79, -0.16 | -3.08 | **0.004** |

ß – regression coefficients, CI – confidence intervals, *p*-values are derived from GLM analysis. Significance values (*p* < 0.05) of BMI and log_10_-transformed biomarker levels (marked in bold) in patients group before and after 7-month treatment.

**Table S-7. Comparison of serum levels of amino acids (γmoles) between the first-episode psychosis (FEP) patients (n=36) at follow-up (FEP_f_) (after 7-month treatment with antipsychotics) and control subjects (CSs) (n=37).**

| *Biomarkers* | FEP_f_ | CSs | Z-value | *p*-value |
| --- | --- | --- | --- | --- |
|  | Median  (min – max) | Median  (min – max) |  |  |
| Alanine (Ala) | 418  (294 – 750) | 405  (232 – 716) | 0.95 | 0.34 |
| Arginine (Arg) | 153  (93.0 – 218) | 152  (94.00 – 225.00) | -1.01 | 0.31 |
| Asparagine (Asn) | 37.9  (17.7 – 75.5) | 33.9  (15.0 – 60.4) | 0.33 | 0.74 |
| Aspartate (Asp) | 29.0  (17.7 – 57.4) | 34.2  (15.9 – 65.2) | -1.56 | 0.12 |
| Citrulline (Citr) | 24.6  (15.5 – 38.7) | 27.4  (11.0 – 48.9) | -1.64 | 0.10 |
| Glutamine (Gln) | 373  (162 – 810) | 308  (77.0 – 683) | 1.24 | 0.22 |
| Glutamate (Glu) | 207  (57.2 – 498) | 183  (114 – 550) | -0.03 | 0.98 |
| Glycine (Gly) | 267  (149 – 597) | 250  (123 – 443) | 1.36 | 0.17 |
| Histidine (His) | 93.1  (73.3 – 132) | 92.1  (58.3 – 138) | 0.52 | 0.60 |
| Isoleucine (Ile) | 94.9  (43.9 – 190) | 85.4  (50.1 – 179) | 0.74 | 0.46 |
| Leucine (Leu) | 173  (85.5 – 364) | 166  (79.6 – 409) | 0.32 | 0.75 |
| Lysine (Lys) | 207  (103 – 306) | 202  (107 – 309) | 0.08 | 0.93 |
| Methionine (Met) | 12.5  (4.53 – 33.5) | 9.08  (4.43 – 35.2) | 2.27 | 0.02 |
| Ornithine (Orn) | 57.5  (28.4 – 91.9) | 56.8  (23.4 – 91.4) | 0.33 | 0.74 |
| Phenylalanine (Phe) | 65.6  (38.2 – 108) | 67.1  (38.2 – 115) | -0.29 | 0.77 |
| Proline (Pro) | 236  (140 – 362) | 215  (123 – 479) | 0.99 | 0.32 |
| Serine (Ser) | 158  (115 – 246) | 160  (69.3 – 363) | -0.12 | 0.90 |
| Threonine (Thr) | 148  (71.0 – 280) | 154  (74.1 – 373) | -0.20 | 0.84 |
| Tryptophan (Trp) | 70.5  (34.2 – 121) | 73.2  (32.8 – 120) | -0.73 | 0.46 |
| Tyrosine (Tyr) | 63.3  (40.6 – 121) | 63.2  (33.7 – 159) | -0.19 | 0.85 |
| Valine (Val) | 231.5  (136 – 390) | 220  (126 – 401) | 0.48 | 0.63 |
| Citr/Arg | 0.17  (0.10 – 0.31) | 0.16  (0.08 – 0.33) | -0.26 | 0.80 |
| Tyr/Phe | 1.01  (0.77 – 1.44) | 1.03  (0.49 – 1.57) | -0.14 | 0.89 |

Z-adjusted values according to Mann-Whitney *U*-test (FEP_f_ compared to CSs). *p-*values less than or equal to 0.001 after Bonferroni correction are marked in bold.

Commentary: all measured values are higher than LLOQ.

**Table S-8. Comparison of serum levels of biogenic amines (γmoles) between the first-episode psychosis (FEP) patients (n=36) at follow-up (FEP_f_) (after 7-month treatment with antipsychotics) and control subjects (CSs) (n=37).**

| *Biomarkers* | FEP_f_ | CSs | Z-value | *p*-value |
| --- | --- | --- | --- | --- |
|  | Median  (min – max) | Median  (min – max) |  |  |
| Acetylornithine  (Ac-Orn) | 0.61  (0.24 – 1.47) | 0.59  (0.18 – 2.03) | 0.25 | 0.80 |
| Asymmetric  Dimethylarginine (ADMA) | 0.41  (0.29 – 0.61) | 0.43  (0.19 – 0.60) | -0.47 | 0.64 |
| Alpha-aminoadipic-acid (alpha-AAA) | 0.81  (0.33 – 1.54) | 0.76  (0.45 – 1.98) | 0.00 | 1.0 |
| c4-OH-Pro | 0.00  (0.00 – 0.38) | 0.00  (0.00 – 0.39) | 0.08 | 0.94 |
| Carnosine | 0.00  (0.00 – 0.15) | 0.00  (0.00 – 0.12) | -2.02 | 0.04 |
| Creatinine | 71.7  (45.7 – 124) | 68.5  (35.0 – 112) | 0.49 | 0.63 |
| l-DOPA | 0.14  (0.00 – 0.30) | 0.15  (0.00 – 0.26) | 0.17 | 0.86 |
| Kynurenine (Kyn) | 2.86  (1.77 – 4.74) | 2.70  (1.37 – 3.89) | 1.70 | 0.09 |
| Histamine | 0.38  (0.37 – 0.45) | 0.38  (0.37 – 0.46) | -0.10 | 0.92 |
| Methionine-sulfoxide  (Met-SO) | 8.72  (1.69 – 20.3) | 10.8  (3.04 – 23.1) | -2.26 | 0.02 |
| Putrescine | 0.07  (0.03 – 0.21) | 0.08  (0.03 – 0.20) | -1.73 | 0.08 |
| Symmetric-dimethylarginine  (S-DMA) | 0.52  (0.39 – 0.80) | 0.53  (0.39 – 0.81) | -0.03 | 0.97 |
| Serotonin (5-HT) | 0.58  (0.05 – 1.33) | 0.65  (0.19 – 1.47) | -1.83 | 0.07 |
| Spermine | 0.19  (0.16 – 0.27) | 0.23  (0.16 – 0.28) | 0.06 | 0.95 |
| t4-OH-Pro | 0.60  (0.00 – 27.1) | 0.61  (0.00 – 20.7) | -0.29 | 0.77 |
| Taurine | 46.6  (28.2 – 119) | 47.1  (25.8 – 116) | 0.19 | 0.85 |
| total-DMA | 0.73  (0.52 – 0.98) | 0.73  (0.37 – 0.96) | 0.26 | 0.79 |
| Met-SO /  Methionine (Met) | 0.66  (0.05 – 3.55) | 1.44  (0.16 – 4.29) | -2.43 | 0.02 |
| Kyn/Tryptophan (Trp) | 0.04  (0.03 – 0.06) | 0.04  (0.03 – 0.05) | 3.09 | 0.002 |
| 5-HT/ Trp | 0.01  (0.00 – 0.02) | 0.01  (0.00 – 0.02) | -1.05 | 0.29 |

Z-adjusted values according to Mann-Whitney *U*-test (FEP_f_ compared to CSs). *p-*values less than or equal to 0.001 after Bonferroni correction are marked in bold. Commentary: ADMA, creatinine, Kyn, Met-So, 5-HT, spermine, taurine, and total-DMA values are higher than LLOQ. Ac-Orn, alpha-AAA, histamine, S-DMA values were at least 1.5 to 3 times higher than LOD.

**Table S-9. Main effect of treatment on serum levels of amino acids and biogenic amines (antipsychotic treated first-episode psychosis patients compared to control subjects).**

| *Biomarkers* | ß | ß (95 % CI) | *t-value* | *p*-value |
| --- | --- | --- | --- | --- |
| Alanine (Ala) | 0.06 | -0.23, 0.35 | 0.42 | 0.67 |
| Arginine (Arg) | -0.18 | -0.47, 0.12 | -1.19 | 0.24 |
| Asparagine (Asn) | 0.13 | -0.17, 0.42 | 0.85 | 0.40 |
| Aspartate (Asp) | -0.23 | -0.52, 0.07 | -1.53 | 0.13 |
| Citrulline (Citr) | -0.05 | -0.35, 0.25 | -0.33 | 0.74 |
| Glutamine (Gln) | 0.31 | 0.03, 0.59 | 2.21 | **0.03** |
| Glutamate (Glu) | -0.20 | -0.46, 0.06 | -1.57 | 0.12 |
| Glycine (Gly) | 0.25 | -0.04, 0.54 | 1.74 | 0.09 |
| Histidine (His) | 0.05 | -0.25, 0.35 | 0.35 | 0.73 |
| Isoleucine (Ile) | -0.11 | -0.38, 0.16 | -0.82 | 0.42 |
| Leucine (Leu) | -0.12 | -0.38, 0.14 | -0.93 | 0.36 |
| Lysine (Lys) | 0.00 | -0.30, 0.30 | 0.00 | 1.00 |
| Methionine (Met) | 0.32 | 0.04, 0.59 | 2.34 | **0.02** |
| Ornithine (Orn) | 0.12 | -0.18, 0.42 | 0.81 | 0.42 |
| Phenylalanine (Phe) | -0.10 | -0.39, 0.20 | -0.67 | 0.51 |
| Proline (Pro) | 0.07 | -0.23, 0.36 | 0.44 | 0.66 |
| Serine (Ser) | 0.11 | -0.19, 0.41 | 0.75 | 0.46 |
| Threonine (Thr) | -0.07 | -0.38, 0.23 | -0.50 | 0.62 |
| Tryptophan (Trp) | -0.10 | -0.39, 0.19 | -0.70 | 0.48 |
| Tyrosine (Tyr) | -0.13 | -0.42, 0.16 | -0.91 | 0.37 |
| Valine (Val) | -0.05 | -0.33, 0.24 | -0.33 | 0.74 |
| Acetylornithine (Ac-Orn) | 0.00 | -0.30, 0.30 | -0.01 | 1.00 |
| Asymmetric dimethylarginine (ADMA) | 0.05 | -0.26, 0.35 | 0.30 | 0.77 |
| Alpha-Aminoadipic-acid (alpha-AAA) | -0.11 | -0.39, 0.18 | -0.75 | 0.46 |
| Creatinine | 0.03 | -0.23, 0.28 | 0.20 | 0.84 |
| L-DOPA | 0.06 | -0.24, 0.35 | 0.38 | 0.71 |
| Kynurenine (Kyn) | 0.25 | -0.01, 0.52 | 1.94 | 0.06 |
| Histamine | -0.15 | -0.45, 0.14 | -1.04 | 0.30 |
| Methioninesulfoxide (Met-SO) | -0.26 | -0.54, 0.02 | -1.84 | 0.07 |
| Putrescine | -0.26 | -0.55, 0.04 | -1.75 | 0.09 |
| Symmetric-dimethylarginine (S-DMA) | -0.02 | -0.31, 0.28 | -0.12 | 0.90 |
| Serotonin (5-HT) | -0.22 | -0.50, 0.07 | -1.52 | 0.13 |
| Spermine | -0.14 | -0.44, 0.15 | -0.97 | 0.34 |
| Taurine | -0.03 | -0.33, 0.28 | -0.18 | 0.86 |
| total-DMA | 0.07 | -0.22, 0.36 | 0.48 | 0.64 |

ß – regression coefficients, CI – confidence intervals, p-values (derived from GLM analysis) – significance values of log_10_-transformed amino acids and biogenic amines levels with treatment condition, adjusted for gender, smoking status, age and BMI. Significant *t*-values (*p* < 0.05) are marked in bold.
