# Supplementary material for: Profiling of Amino Acids and Their Derivatives Biogenic Amines Before and After Antipsychotic Treatment in First-Episode Psychosis
Source: Front Psychiatry. 2018 Apr 24;9:155. doi: 10.3389/fpsyt.2018.00155 (PMC5928450; doi:10.3389/fpsyt.2018.00155)
Supplement: Supplementary file 4 [file Table_4.DOCX]

***Supplementary Material***

**Profiling of Amino Acids and their Derivatives Biogenic Amines Before and After Antipsychotic Treatment in First-Episode Psychosis**

Liisa Leppik^a,b*^, Kärt Kriisa^a^, Kati Koido^a^, Kadri Koch^a,b^, Kärolin Kajalaid^a,b^, Liina Haring^a,b,c^, Eero Vasar^a,c^, Mihkel Zilmer^a,c^

^a^ − Institute of Biomedicine and Translational Medicine, University of Tartu, Tartu, Estonia

^b^ − Psychiatry Clinic of Tartu University Hospital, Tartu, Estonia

^c^ − contribution of these authors has been equal

^*^ − corresponding author Liisa Leppik [liisa.leppik@kliinikum.ee](mailto:liisa.leppik@kliinikum.ee)

**Table S-4. Comparison of serum levels of amino acids (γmoles) between the first-episode psychosis (FEP) patients (n=36) at baseline (before treatment with antipsychotics, FEP_b_) and after 7-month treatment (FEP_f_) (n=36) with antipsychotics.**

| *Amino acids* | FEP_b_ | FEP_f_ | Z-value | *p*-value |
| --- | --- | --- | --- | --- |
|  | Median  (min–max) | Median  (min–max) |  |  |
| Alanine (Ala) | 342.5  (206.00 – 673.00) | 418  (294 – 750) | 2.66 | 0.008 |
| Arginine (Arg) | 148  (88.0 – 216) | 153  (93.0 – 218) | 0.44 | 0.66 |
| Asparagine (Asn) | 36.8  (19.5 – 83.3) | 37.9  (17.7 – 75.5) | 1.21 | 0.23 |
| Aspartate (Asp) | 38.7  (18.8 – 62.9) | 29.0  (17.7 – 57.4) | 2.50 | 0.01 |
| Citrulline (Citr) | 22.4  (12.6 – 38.1) | 24.6  (15.5 – 38.7) | 1.54 | 0.12 |
| Glutamine (Gln) | 377  (118 – 813) | 372.5  (162 – 810) | 1.52 | 0.13 |
| Glutamate (Glu) | 210  (59.6 – 381) | 207  (57.2 – 498) | 1.17 | 0.24 |
| Glycine (Gly) | 274  (153 – 420) | 267  (149 – 597) | 0.23 | 0.82 |
| Histidine (His) | 82.6  (61.5 – 106) | 93.1  (73.3 – 132) | 3.75 | **0.0002** |
| Isoleucine (Ile) | 85.0  (42.7 – 130) | 94.9  (43.9 – 190) | 1.78 | 0.07 |
| Leucine (Leu) | 165  (73.0 – 273) | 173  (85.5 – 364) | 0.41 | 0.68 |
| Lysine (Lys) | 183.5  (117 – 279) | 207  (103 – 306) | 1.47 | 0.14 |
| Methionine (Met) | 7.75  (4.46 – 26.3) | 12.5  (4.53 – 33.5) | 2.50 | 0.01 |
| Ornithine (Orn) | 57.3  (30.7 – 115) | 57.5  (28.4 – 91.9) | 0.90 | 0.37 |
| Phenylalanine (Phe) | 72.0  (41.8 – 101) | 65.6  (38.2 – 108) | 1.35 | 0.18 |
| Proline (Pro) | 166  (83.3 – 381) | 236  (140 – 362) | 4.15 | **<0.0001** |
| Serine (Ser) | 171  (99.4 – 293) | 158  (115 – 246) | 1.18 | 0.24 |
| Threonine (Thr) | 140  (84.7 – 214) | 148  (71.0 – 280) | 1.71 | 0.09 |
| Tryptophan (Trp) | 64.8  (30.3 – 89.3) | 70.5  (34.2 – 121) | 1.68 | 0.09 |
| Tyrosine (Tyr) | 58.6  (35.8 – 88.7) | 63.3  (40.6 – 121) | 2.99 | 0.003 |
| Valine (Val) | 198  (112 – 299) | 232  (136 – 390) | 2.92 | 0.003 |
| Citr/Arg | 0.16  (0.09 – 0.31) | 0.17  (0.10 – 0.31) | 1.81 | 0.07 |
| Tyr/Phe | 0.82  (0.61 – 1.26) | 1.01  (0.77 – 1.44) | 4.46 | **<0.0001** |

Z-values according to Wilcoxon Matched Pairs Test (FEP_b_ compared to FEP_f_). *p-*values less than or equal to 0.001 after Bonferroni correction are marked in bold. Commentary: all measured values are higher than LLOQ.
