# Supplementary material for: Profiling of Amino Acids and Their Derivatives Biogenic Amines Before and After Antipsychotic Treatment in First-Episode Psychosis
Source: Front Psychiatry. 2018 Apr 24;9:155. doi: 10.3389/fpsyt.2018.00155 (PMC5928450; doi:10.3389/fpsyt.2018.00155)
Supplement: Supplementary file 5 [file Table_5.DOCX]

***Supplementary Material***

**Profiling of Amino Acids and their Derivatives Biogenic Amines Before and After Antipsychotic Treatment in First-Episode Psychosis**

Liisa Leppik^a,b*^, Kärt Kriisa^a^, Kati Koido^a^, Kadri Koch^a,b^, Kärolin Kajalaid^a,b^, Liina Haring^a,b,c^, Eero Vasar^a,c^, Mihkel Zilmer^a,c^

^a^ − Institute of Biomedicine and Translational Medicine, University of Tartu, Tartu, Estonia

^b^ − Psychiatry Clinic of Tartu University Hospital, Tartu, Estonia

^c^ − contribution of these authors has been equal

^*^ − corresponding author Liisa Leppik [liisa.leppik@kliinikum.ee](mailto:liisa.leppik@kliinikum.ee)

**Table S-5. Comparison of serum levels of biogenic amines (γmoles) between the first-episode psychosis (FEP) patients (n=36) at baseline (FEP_b_) (before treatment with antipsychotic) and after 7-month treatment (FEP_f_) (n=36) with antipsychotics.**

| *Biogenic amines* | FEP_b_ | FEP_f_ | Z-value | *p*-value |
| --- | --- | --- | --- | --- |
|  | Median  (min – max) | Median  (min – max) |  |  |
| Acetylornithine  (Ac-Orn) | 0.56  (0.18 – 1.06) | 0.61  (0.24 – 1.47) | 3.41 | **0.0007** |
| Asymmetric  dimethylarginine (ADMA) | 0.43  (0.30 – 0.67) | 0.41  (0.29 – 0.61) | 0.20 | 0.84 |
| Alpha aminoadipic acid (alpha-AAA) | 0.56  (0.25 – 1.34) | 0.81  (0.33 – 1.54) | 3.33 | **0.0009** |
| c4-OH-Pro | 0.25  (0.00 – 0.34) | 0.00  (0.00 – 0.38) | 1.72 | 0.09 |
| Carnosine | 0.00  (0.00 – 0.13) | 0.00  (0.00 – 0.15) | 2.03 | 0.04 |
| Creatinine | 69.7  (42.3 – 123) | 71.7  (45.7 – 124) | 0.03 | 0.98 |
| l-DOPA | 0.12  (0.00 – 0.26) | 0.14  (0.00 – 0.30) | 1.05 | 0.30 |
| Kynurenine (Kyn) | 2.20  (1.39 – 5.42) | 2.86  (1.77 – 4.74) | 3.59 | **0.0003** |
| Histamine | 0.45  (0.37 – 0.46) | 0.38  (0.37 – 0.45) | 1.89 | 0.06 |
| Methioninesulfoxide  (Met-SO) | 10.4  (2.11 – 24.9) | 8.72  (1.69 – 20.3) | 2.05 | 0.04 |
| Putrescine | 0.07  (0.02 – 0.19) | 0.07  (0.03 – 0.21) | 0.07 | 0.94 |
| Symmetric-dimethylarginine  (S-DMA) | 0.57  (0.39 – 0.93) | 0.52  (0.39 – 0.80) | 1.34 | 0.18 |
| Serotonin (5-HT) | 0.57  (0.08 – 1.69) | 0.58  (0.05 – 1.33) | 1.56 | 0.12 |
| Spermine | 0.27  (0.17 – 0.43) | 0.19  (0.16 – 0.27) | 2.79 | 0.005 |
| t4-OH-Pro | 0.42  (0.00 – 15.30) | 0.60  (0.00 – 27.10) | 0.51 | 0.61 |
| Taurine | 76.5  (32.4 – 172) | 46.6  (28.2 – 119) | 5.17 | **<0.0001** |
| total-DMA | 0.70  (0.49 – 1.08) | 0.73  (0.52 – 0.98) | 0.50 | 0.62 |
| Met-SO/  Methionine (Met) | 1.35  (0.11 – 4.39) | 0.66  (0.05 – 3.55) | 2.14 | 0.03 |
| Kyn/Tryptophan (Trp) | 0.03  (0.02 – 0.08) | 0.04  (0.03 – 0.06) | 2.70 | 0.007 |
| 5-HT/Trp | 0.01  (0.00 – 0.03) | 0.01  (0.00 – 0.02) | 1.17 | 0.24 |

Z-values according to Wilcoxon Matched Pairs Test (FEP_b_ compared to FEP_f_). *p-*values less than or equal to 0.001 after Bonferroni correction are marked in bold. Commentary: ADMA, creatinine, Kyn, Met-So, 5-HT, spermine, taurine, and total-DMA values are higher than LLOQ. Ac-Orn, alpha-AAA, histamine, S-DMA values were at least 1.5 to 3 times higher than LOD.
