# Supplementary material for: Profiling of Amino Acids and Their Derivatives Biogenic Amines Before and After Antipsychotic Treatment in First-Episode Psychosis
Source: Front Psychiatry. 2018 Apr 24;9:155. doi: 10.3389/fpsyt.2018.00155 (PMC5928450; doi:10.3389/fpsyt.2018.00155)
Supplement: Supplementary file 6 [file Table_6.DOCX]

***Supplementary Material***

**Profiling of Amino Acids and their Derivatives Biogenic Amines Before and After Antipsychotic Treatment in First-Episode Psychosis**

Liisa Leppik^a,b*^, Kärt Kriisa^a^, Kati Koido^a^, Kadri Koch^a,b^, Kärolin Kajalaid^a,b^, Liina Haring^a,b,c^, Eero Vasar^a,c^, Mihkel Zilmer^a,c^

^a^ − Institute of Biomedicine and Translational Medicine, University of Tartu, Tartu, Estonia

^b^ − Psychiatry Clinic of Tartu University Hospital, Tartu, Estonia

^c^ − contribution of these authors has been equal

^*^ − corresponding author Liisa Leppik [liisa.leppik@kliinikum.ee](mailto:liisa.leppik@kliinikum.ee)

**Table S-6. Main effect of the antipsychotic treatment on serum levels of amino acids and biogenic amines and body mass index (BMI), first-episode psychosis patients data compared before and after 7-month treatment with antipsychotics.**

| *Biomarkers and BMI* | ß | ß (95 % CI) | *t-value* | *p*-value |
| --- | --- | --- | --- | --- |
| Alanine (Ala) | -0.35 | -0.63, -0.06 | -2.45 | **0.02** |
| Arginine (Arg) | 0.05 | -0.30, 0.40 | 0.31 | 0.76 |
| Asparagine (Asn) | -0.05 | -0.39, 0.29 | -0.29 | 0.78 |
| Aspartate (Asp) | 0.42 | 0.10, 0.75 | 2.60 | **0.01** |
| Citrulline (Citr) | -0.11 | -0.44, 0.22 | -0.66 | 0.51 |
| Glutamine (Gln) | -0.19 | -0.52, 0.14 | -1.16 | 0.25 |
| Glutamate (Glu) | 0.16 | -0.16, 0.49 | 1.00 | 0.32 |
| Glycine (Gly) | 0.03 | -0.32, 0.37 | 0.15 | 0.88 |
| Histidine (His) | -0.39 | -0.69, -0.09 | -2.60 | **0.01** |
| Isoleucine (Ile) | -0.17 | -0.51, 0.17 | -1.00 | 0.32 |
| Leucine (Leu) | -0.07 | -0.41, 0.28 | -0.38 | 0.70 |
| Lysine (Lys) | -0.05 | -0.40, 0.29 | -0.32 | 0.75 |
| Methionine (Met) | -0.21 | -0.55, 0.12 | -1.30 | 0.20 |
| Ornithine (Orn) | 0.15 | -0.20, 0.49 | 0.86 | 0.40 |
| Phenylalanine (Phe) | 0.13 | -0.21, 0.48 | 0.78 | 0.44 |
| Proline (Pro) | -0.55 | -0.79, -0.31 | -4.55 | **0.00004** |
| Serine (Ser) | 0.22 | -0.11, 0.54 | 1.35 | 0.18 |
| Threonine (Thr) | -0.16 | -0.50, 0.19 | -0.92 | 0.36 |
| Tryptophan (Trp) | -0.15 | -0.49, 0.19 | -0.90 | 0.37 |
| Tyrosine (Tyr) | -0.29 | -0.61, 0.03 | -1.84 | 0.07 |
| Valine (Val) | -0.26 | -0.58, 0.05 | -1.67 | 0.10 |
| Acetylornithine (Ac-Orn) | -0.10 | -0.45, 0.24 | -0.61 | 0.54 |
| Asymmetric dimethylarginine (ADMA) | 0.14 | -0.20, 0.48 | 0.82 | 0.41 |
| Alpha-Aminoadipic-acid (alpha-AAA) | -0.39 | -0.70, -0.08 | -2.51 | **0.02** |
| Creatinine | 0.09 | -0.24, 0.42 | 0.54 | 0.59 |
| L-DOPA | 0.09 | -0.24, 0.42 | 0.54 | 0.59 |
| Kynurenine (Kyn) | -0.35 | -0.65, -0.06 | -2.40 | **0.02** |
| Histamine | 0.21 | -0.13, 0.54 | 1.25 | 0.22 |
| Methionine-sulfoxide (Met-SO) | 0.14 | -0.19, 0.48 | 0.84 | 0.40 |
| Putrescine | 0.12 | -0.22, 0.46 | 0.70 | 0.49 |
| Symmetric-dimethylarginine (S-DMA) | 0.26 | -0.08, 0.60 | 1.56 | 0.13 |
| Serotonin (5-HT) | 0.13 | -0.20, 0.47 | 0.79 | 0.43 |
| Spermine | 0.25 | -0.08, 0.58 | 1.52 | 0.14 |
| Taurine | 0.70 | 0.46, 0.94 | 5.88 | **0.0000005** |
| total-DMA | 0.02 | -0.32, 0.37 | 0.13 | 0.90 |
| BMI | -0.48 | -0.79, -0.16 | -3.08 | **0.004** |

ß – regression coefficients, CI – confidence intervals, *p*-values are derived from GLM analysis. Significance values (*p* < 0.05) of BMI and log_10_-transformed biomarker levels (marked in bold) in patients group before and after 7-month treatment.
