# Supplementary material for: Profiling of Amino Acids and Their Derivatives Biogenic Amines Before and After Antipsychotic Treatment in First-Episode Psychosis
Source: Front Psychiatry. 2018 Apr 24;9:155. doi: 10.3389/fpsyt.2018.00155 (PMC5928450; doi:10.3389/fpsyt.2018.00155)
Supplement: Supplementary file 7 [file Table_7.DOCX]

***Supplementary Material***

**Profiling of Amino Acids and their Derivatives Biogenic Amines Before and After Antipsychotic Treatment in First-Episode Psychosis**

Liisa Leppik^a,b*^, Kärt Kriisa^a^, Kati Koido^a^, Kadri Koch^a,b^, Kärolin Kajalaid^a,b^, Liina Haring^a,b,c^, Eero Vasar^a,c^, Mihkel Zilmer^a,c^

^a^ − Institute of Biomedicine and Translational Medicine, University of Tartu, Tartu, Estonia

^b^ − Psychiatry Clinic of Tartu University Hospital, Tartu, Estonia

^c^ − contribution of these authors has been equal

^*^ − corresponding author Liisa Leppik [liisa.leppik@kliinikum.ee](mailto:liisa.leppik@kliinikum.ee)

**Table S-7. Comparison of serum levels of amino acids (γmoles) between the first-episode psychosis (FEP) patients (n=36) at follow-up (FEP_f_) (after 7-month treatment with antipsychotics) and control subjects (CSs) (n=37).**

| *Biomarkers* | FEP_f_ | CSs | Z-value | *p*-value |
| --- | --- | --- | --- | --- |
|  | Median  (min – max) | Median  (min – max) |  |  |
| Alanine (Ala) | 418  (294 – 750) | 405  (232 – 716) | 0.95 | 0.34 |
| Arginine (Arg) | 153  (93.0 – 218) | 152  (94.00 – 225.00) | -1.01 | 0.31 |
| Asparagine (Asn) | 37.9  (17.7 – 75.5) | 33.9  (15.0 – 60.4) | 0.33 | 0.74 |
| Aspartate (Asp) | 29.0  (17.7 – 57.4) | 34.2  (15.9 – 65.2) | -1.56 | 0.12 |
| Citrulline (Citr) | 24.6  (15.5 – 38.7) | 27.4  (11.0 – 48.9) | -1.64 | 0.10 |
| Glutamine (Gln) | 373  (162 – 810) | 308  (77.0 – 683) | 1.24 | 0.22 |
| Glutamate (Glu) | 207  (57.2 – 498) | 183  (114 – 550) | -0.03 | 0.98 |
| Glycine (Gly) | 267  (149 – 597) | 250  (123 – 443) | 1.36 | 0.17 |
| Histidine (His) | 93.1  (73.3 – 132) | 92.1  (58.3 – 138) | 0.52 | 0.60 |
| Isoleucine (Ile) | 94.9  (43.9 – 190) | 85.4  (50.1 – 179) | 0.74 | 0.46 |
| Leucine (Leu) | 173  (85.5 – 364) | 166  (79.6 – 409) | 0.32 | 0.75 |
| Lysine (Lys) | 207  (103 – 306) | 202  (107 – 309) | 0.08 | 0.93 |
| Methionine (Met) | 12.5  (4.53 – 33.5) | 9.08  (4.43 – 35.2) | 2.27 | 0.02 |
| Ornithine (Orn) | 57.5  (28.4 – 91.9) | 56.8  (23.4 – 91.4) | 0.33 | 0.74 |
| Phenylalanine (Phe) | 65.6  (38.2 – 108) | 67.1  (38.2 – 115) | -0.29 | 0.77 |
| Proline (Pro) | 236  (140 – 362) | 215  (123 – 479) | 0.99 | 0.32 |
| Serine (Ser) | 158  (115 – 246) | 160  (69.3 – 363) | -0.12 | 0.90 |
| Threonine (Thr) | 148  (71.0 – 280) | 154  (74.1 – 373) | -0.20 | 0.84 |
| Tryptophan (Trp) | 70.5  (34.2 – 121) | 73.2  (32.8 – 120) | -0.73 | 0.46 |
| Tyrosine (Tyr) | 63.3  (40.6 – 121) | 63.2  (33.7 – 159) | -0.19 | 0.85 |
| Valine (Val) | 231.5  (136 – 390) | 220  (126 – 401) | 0.48 | 0.63 |
| Citr/Arg | 0.17  (0.10 – 0.31) | 0.16  (0.08 – 0.33) | -0.26 | 0.80 |
| Tyr/Phe | 1.01  (0.77 – 1.44) | 1.03  (0.49 – 1.57) | -0.14 | 0.89 |

Z-adjusted values according to Mann-Whitney *U*-test (FEP_f_ compared to CSs). *p-*values less than or equal to 0.001 after Bonferroni correction are marked in bold. Commentary: all measured values are higher than LLOQ.
