# Supplementary material for: Profiling of Amino Acids and Their Derivatives Biogenic Amines Before and After Antipsychotic Treatment in First-Episode Psychosis
Source: Front Psychiatry. 2018 Apr 24;9:155. doi: 10.3389/fpsyt.2018.00155 (PMC5928450; doi:10.3389/fpsyt.2018.00155)
Supplement: Supplementary file 8 [file Table_8.DOCX]

***Supplementary Material***

**Profiling of Amino Acids and their Derivatives Biogenic Amines Before and After Antipsychotic Treatment in First-Episode Psychosis**

Liisa Leppik^a,b*^, Kärt Kriisa^a^, Kati Koido^a^, Kadri Koch^a,b^, Kärolin Kajalaid^a,b^, Liina Haring^a,b,c^, Eero Vasar^a,c^, Mihkel Zilmer^a,c^

^a^ − Institute of Biomedicine and Translational Medicine, University of Tartu, Tartu, Estonia

^b^ − Psychiatry Clinic of Tartu University Hospital, Tartu, Estonia

^c^ − contribution of these authors has been equal

^*^ − corresponding author Liisa Leppik [liisa.leppik@kliinikum.ee](mailto:liisa.leppik@kliinikum.ee)

**Table S-8. Comparison of serum levels of biogenic amines (γmoles) between the first-episode psychosis (FEP) patients (n=36) at follow-up (FEP_f_) (after 7-month treatment with antipsychotics) and control subjects (CSs) (n=37).**

| *Biomarkers* | FEP_f_ | CSs | Z-value | *p*-value |
| --- | --- | --- | --- | --- |
|  | Median  (min – max) | Median  (min – max) |  |  |
| Acetylornithine  (Ac-Orn) | 0.61  (0.24 – 1.47) | 0.59  (0.18 – 2.03) | 0.25 | 0.80 |
| Asymmetric  Dimethylarginine (ADMA) | 0.41  (0.29 – 0.61) | 0.43  (0.19 – 0.60) | -0.47 | 0.64 |
| Alpha-aminoadipic-acid (alpha-AAA) | 0.81  (0.33 – 1.54) | 0.76  (0.45 – 1.98) | 0.00 | 1.0 |
| c4-OH-Pro | 0.00  (0.00 – 0.38) | 0.00  (0.00 – 0.39) | 0.08 | 0.94 |
| Carnosine | 0.00  (0.00 – 0.15) | 0.00  (0.00 – 0.12) | -2.02 | 0.04 |
| Creatinine | 71.7  (45.7 – 124) | 68.5  (35.0 – 112) | 0.49 | 0.63 |
| l-DOPA | 0.14  (0.00 – 0.30) | 0.15  (0.00 – 0.26) | 0.17 | 0.86 |
| Kynurenine (Kyn) | 2.86  (1.77 – 4.74) | 2.70  (1.37 – 3.89) | 1.70 | 0.09 |
| Histamine | 0.38  (0.37 – 0.45) | 0.38  (0.37 – 0.46) | -0.10 | 0.92 |
| Methionine-sulfoxide  (Met-SO) | 8.72  (1.69 – 20.3) | 10.8  (3.04 – 23.1) | -2.26 | 0.02 |
| Putrescine | 0.07  (0.03 – 0.21) | 0.08  (0.03 – 0.20) | -1.73 | 0.08 |
| Symmetric-dimethylarginine  (S-DMA) | 0.52  (0.39 – 0.80) | 0.53  (0.39 – 0.81) | -0.03 | 0.97 |
| Serotonin (5-HT) | 0.58  (0.05 – 1.33) | 0.65  (0.19 – 1.47) | -1.83 | 0.07 |
| Spermine | 0.19  (0.16 – 0.27) | 0.23  (0.16 – 0.28) | 0.06 | 0.95 |
| t4-OH-Pro | 0.60  (0.00 – 27.1) | 0.61  (0.00 – 20.7) | -0.29 | 0.77 |
| Taurine | 46.6  (28.2 – 119) | 47.1  (25.8 – 116) | 0.19 | 0.85 |
| total-DMA | 0.73  (0.52 – 0.98) | 0.73  (0.37 – 0.96) | 0.26 | 0.79 |
| Met-SO /  Methionine (Met) | 0.66  (0.05 – 3.55) | 1.44  (0.16 – 4.29) | -2.43 | 0.02 |
| Kyn/Tryptophan (Trp) | 0.04  (0.03 – 0.06) | 0.04  (0.03 – 0.05) | 3.09 | 0.002 |
| 5-HT/ Trp | 0.01  (0.00 – 0.02) | 0.01  (0.00 – 0.02) | -1.05 | 0.29 |

Z-adjusted values according to Mann-Whitney *U*-test (FEP_f_ compared to CSs). *p-*values less than or equal to 0.001 after Bonferroni correction are marked in bold. Commentary: ADMA, creatinine, Kyn, Met-So, 5-HT, spermine, taurine, and total-DMA values are higher than LLOQ. Ac-Orn, alpha-AAA, histamine, S-DMA values were at least 1.5 to 3 times higher than LOD.
