# Supplementary material for: Profiling of Amino Acids and Their Derivatives Biogenic Amines Before and After Antipsychotic Treatment in First-Episode Psychosis
Source: Front Psychiatry. 2018 Apr 24;9:155. doi: 10.3389/fpsyt.2018.00155 (PMC5928450; doi:10.3389/fpsyt.2018.00155)
Supplement: Supplementary file 9 [file Table_9.DOCX]

***Supplementary Material***

**Profiling of Amino Acids and their Derivatives Biogenic Amines Before and After Antipsychotic Treatment in First-Episode Psychosis**

Liisa Leppik^a,b*^, Kärt Kriisa^a^, Kati Koido^a^, Kadri Koch^a,b^, Kärolin Kajalaid^a,b^, Liina Haring^a,b,c^, Eero Vasar^a,c^, Mihkel Zilmer^a,c^

^a^ − Institute of Biomedicine and Translational Medicine, University of Tartu, Tartu, Estonia

^b^ − Psychiatry Clinic of Tartu University Hospital, Tartu, Estonia

^c^ − contribution of these authors has been equal

^*^ − corresponding author Liisa Leppik [liisa.leppik@kliinikum.ee](mailto:liisa.leppik@kliinikum.ee)

**Table S-9. Main effect of treatment on serum levels of amino acids and biogenic amines (antipsychotic treated first-episode psychosis patients compared to control subjects).**

| *Biomarkers* | ß | ß (95 % CI) | *t-value* | *p*-value |
| --- | --- | --- | --- | --- |
| Alanine (Ala) | 0.06 | -0.23, 0.35 | 0.42 | 0.67 |
| Arginine (Arg) | -0.18 | -0.47, 0.12 | -1.19 | 0.24 |
| Asparagine (Asn) | 0.13 | -0.17, 0.42 | 0.85 | 0.40 |
| Aspartate (Asp) | -0.23 | -0.52, 0.07 | -1.53 | 0.13 |
| Citrulline (Citr) | -0.05 | -0.35, 0.25 | -0.33 | 0.74 |
| Glutamine (Gln) | 0.31 | 0.03, 0.59 | 2.21 | **0.03** |
| Glutamate (Glu) | -0.20 | -0.46, 0.06 | -1.57 | 0.12 |
| Glycine (Gly) | 0.25 | -0.04, 0.54 | 1.74 | 0.09 |
| Histidine (His) | 0.05 | -0.25, 0.35 | 0.35 | 0.73 |
| Isoleucine (Ile) | -0.11 | -0.38, 0.16 | -0.82 | 0.42 |
| Leucine (Leu) | -0.12 | -0.38, 0.14 | -0.93 | 0.36 |
| Lysine (Lys) | 0.00 | -0.30, 0.30 | 0.00 | 1.00 |
| Methionine (Met) | 0.32 | 0.04, 0.59 | 2.34 | **0.02** |
| Ornithine (Orn) | 0.12 | -0.18, 0.42 | 0.81 | 0.42 |
| Phenylalanine (Phe) | -0.10 | -0.39, 0.20 | -0.67 | 0.51 |
| Proline (Pro) | 0.07 | -0.23, 0.36 | 0.44 | 0.66 |
| Serine (Ser) | 0.11 | -0.19, 0.41 | 0.75 | 0.46 |
| Threonine (Thr) | -0.07 | -0.38, 0.23 | -0.50 | 0.62 |
| Tryptophan (Trp) | -0.10 | -0.39, 0.19 | -0.70 | 0.48 |
| Tyrosine (Tyr) | -0.13 | -0.42, 0.16 | -0.91 | 0.37 |
| Valine (Val) | -0.05 | -0.33, 0.24 | -0.33 | 0.74 |
| Acetylornithine (Ac-Orn) | 0.00 | -0.30, 0.30 | -0.01 | 1.00 |
| Asymmetric dimethylarginine (ADMA) | 0.05 | -0.26, 0.35 | 0.30 | 0.77 |
| Alpha-Aminoadipic-acid (alpha-AAA) | -0.11 | -0.39, 0.18 | -0.75 | 0.46 |
| Creatinine | 0.03 | -0.23, 0.28 | 0.20 | 0.84 |
| L-DOPA | 0.06 | -0.24, 0.35 | 0.38 | 0.71 |
| Kynurenine (Kyn) | 0.25 | -0.01, 0.52 | 1.94 | 0.06 |
| Histamine | -0.15 | -0.45, 0.14 | -1.04 | 0.30 |
| Methioninesulfoxide (Met-SO) | -0.26 | -0.54, 0.02 | -1.84 | 0.07 |
| Putrescine | -0.26 | -0.55, 0.04 | -1.75 | 0.09 |
| Symmetric-dimethylarginine (S-DMA) | -0.02 | -0.31, 0.28 | -0.12 | 0.90 |
| Serotonin (5-HT) | -0.22 | -0.50, 0.07 | -1.52 | 0.13 |
| Spermine | -0.14 | -0.44, 0.15 | -0.97 | 0.34 |
| Taurine | -0.03 | -0.33, 0.28 | -0.18 | 0.86 |
| total-DMA | 0.07 | -0.22, 0.36 | 0.48 | 0.64 |

ß – regression coefficients, CI – confidence intervals, p-values (derived from GLM analysis) – significance values of log_10_-transformed amino acids and biogenic amines levels with treatment condition, adjusted for gender, smoking status, age and BMI. Significant *t*-values (*p* < 0.05) are marked in bold.
